# The Burden of Cancer, Government Strategic Policies, and Challenges in Pakistan: A Comprehensive Review

**DOI:** 10.3389/fnut.2022.940514

**Published:** 2022-07-22

**Authors:** Anwar Ali, Muhammad Faisal Manzoor, Nazir Ahmad, Rana Muhammad Aadil, Hong Qin, Rabia Siddique, Sakhawat Riaz, Arslan Ahmad, Sameh A. Korma, Waseem Khalid, Liu Aizhong

**Affiliations:** ^1^Department of Epidemiology and Health Statistics, Xiangya School of Public Health, Central South University, Changsha, China; ^2^Hunan Provincial Key Laboratory of Clinical Epidemiology, Xiangya School of Public Health, Central South University, Changsha, China; ^3^Food and Nutrition Society, Gilgit Baltistan, Pakistan; ^4^School of Food and Biological Engineering, Jiangsu University, Zhenjiang, China; ^5^Department of Nutritional Science, Government College University Faisalabad, Faisalabad, Pakistan; ^6^National Institute of Food Science and Technology, University of Agriculture, Faisalabad, Pakistan; ^7^School of Nutrition and Food Hygiene, Xiangya School of Public Health, Central South University, Changsha, China; ^8^Department of Chemistry, Government College University Faisalabad, Faisalabad, Pakistan; ^9^Department of Home Economics, Government College University Faisalabad, Faisalabad, Pakistan; ^10^Department of Food Science, Faculty of Agriculture, Zagazig, Egypt; ^11^Department of Food Sciences, Government College University, Faisalabad, Pakistan

**Keywords:** cancer epidemiology, healthcare policy, health services, burden of disease, food adulteration

## Abstract

Cancer is a severe condition characterized by uncontrolled cell division and increasing reported mortality and diagnostic cases. In 2040, an estimated 28.4 million cancer cases are expected to happen globally. In 2020, an estimated 19.3 million new cancer cases (18.1 million excluding non-melanoma skin cancer) had been diagnosed worldwide, with around 10.0 million cancer deaths. Breast cancer cases have increased by 2.26 million, lung cancer by 2.21 million, stomach by 1.089 million, liver by 0.96 million, and colon cancer by 1.93 million. Cancer is becoming more prevalent in Pakistan, with 19 million new cancer cases recorded in 2020. Food adulteration, gutkha, paan, and nutritional deficiencies are major cancer risk factors that interplay with cancer pathogenesis in this country. Government policies and legislation, cancer treatment challenges, and prevention must be revised seriously. This review presents the current cancer epidemiology in Pakistan to better understand cancer basis. It summarizes current cancer risk factors, causes, and the strategies and policies of the country against cancer.

## Introduction

Cancer is one of the arduous diseases in which the entry and spread of irregular cell development to other body regions occur. Cancer signs and symptoms depend on cancer grade and type. There are many causes of cancer in which, mostly inherited genetic abnormalities (such as BRCA1 and BRCA2 mutations) (1), infections (2), environmental factors (such as air pollution) (3), and bad lifestyle choices (such as smoking and high alcohol use) (4), may damage DNA and cause cancer. Fatigue, weight loss, skin changes, unusual bleeding, persistent cough, fever, lump, and tissue mass are the common symptoms of this disease (5). Different drug strategies have been developed to treat cancer in different conditions (6). Carcinoma affects the skin, lungs, breast, pancreas, and other organs. Sarcoma is a kind of cancer that affects the joints, muscle, fats, arteries, collagen, and other collagenous tissues of the body. Melanoma occurs in cells that make pigments in the skin (7). Lymphoma is a cancer of lymphocytes and leukemia occurs in the blood. Breast, leukemia, lips, and oral void space cancer are the top 3 tumors in all age groups and gender (8). According to Shaukat Khanum Memorial Cancer Hospital and Research Center Lahore (SKMCH&RC) (9), the three most common cancers among young women are breast, ovary and uterine adnexa, and lip and oral hollow space cancers (9).

The three most common cancers among adult men are genital, intestine canal/anus, and lip and oral hollow space cancers (9). The most common cancers in adults, regardless of gender, are malignant neoplasms of the chest (10), lips and mouth (11), and intestine canal/anus (12). Non-Hodgkin lymphoma (NHL) and Hodgkin lymphoma (HL) are the most frequent cancers in children (13). NHL in children may occur at any age, although it is more frequent in younger children. Adolescents are more likely to get HL (13). In children, there is no recognized cause of lymphoma. Young males with the least frequently diagnosed cancers are stomach, prostate, and mouth (14), while mature girls are commonly diagnosed with chest, cervical, and intestine canal/anus cancers (15).

The 8th edition of the American Joint Committee on Cancer staging recommendations, Tumor nodes and metastasis (TNM), classifies malignancies into four stages from 0 to 4 and unstageable and no longer acceptable tumors, according to those of unstageable tumors to all publicly available cancer websites and registry (16). According to the reports, 0.8% of the 6,291 analytical cases were assigned to stage 0, 12.9% to stage-I, and 27.2% to stage II. There were 25.4% in stage III and 18.2% in stage IV, with 10.2% getting no level and 5.3% implacable (9).

### Mechanisms of Cancers

**Figure 1:**
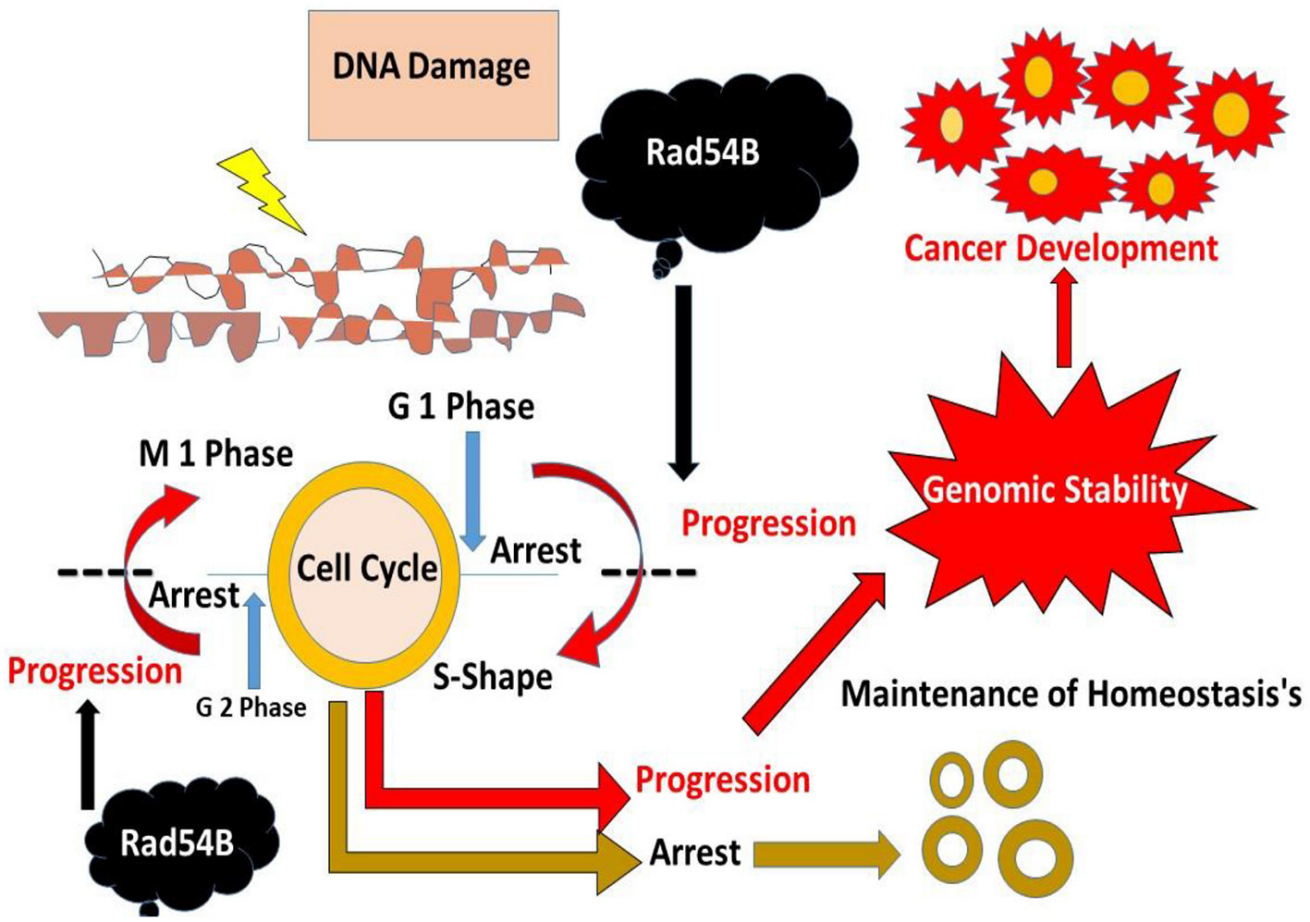
The different mechanisms of cancer and its progression.

The cancer spreads as the normal processes that govern cell activity fail. A single mobile is becoming the father of many cells with unusual abilities or behaviors. It is often the result of cells accumulating genetic damage over time. Sustained proliferative signaling (17), cell death resistance (18), invasion and metastasis activation (19), and angiogenesis induction (20) are substitutes for cancer (21, 22). Figure 1 shows how most cells inside an individual are vulnerable to DNA damage. An individual's cells usually grow and divide in a highly controlled way known as a mobile cycle throughout their lives (23). It helps tissues to mature but also live a healthy life. Until a mobile phase cell divides, it must replicate its DNA (and hence its genetic code) so that each daughter cell has DNA equivalent to the parent cell. DNA replication is a complicated process prone to sequencing errors (24).

Cells are constantly found to have elements that could harm DNA (25). Exogenous factors, such as rays or chemicals in cigarette smoke, and endogenous indicators, such as free radicals or toxic metabolites, are present in the body (26). A carcinogen is a chemical or agent capable of producing cancer, even if not all cancer-causing substances destroy DNA immediately (27). Endogenous defensive mechanisms are less effective due to inherent genetic abnormalities, excessive amounts of exposure to external cancer-causing chemicals, and endogenous elements that damage DNA integrity (27). Unsuitable nutrients over the whole basis represent a disordered dietary microenvironment at the cell level. This can lead to an environment that encourages the buildup of DNA damage and, as a result, the development of cancer stated to the World Cancer Research Fund (WCRF) (22).

## Global Burden of Cancer

Cancer is the leading killer, with more than 10 million deaths in 2020 (28, 29). According to the cancer statistics 2020 (29), breast cancer (2.26 million cases) (29), lung cancer (2.21 million cases) (29), intestine and rectum cancer (1.93 million cases), prostate cancer (1.93 million cases), carcinoma (1.20 million cases), and stomach cancer (1.20 million cases) are the most common cancers in the United States in 2020 (in terms of new cases; 1.09 million cases) (29). The shortage of oxygen in the body is the leading cause of cancer mortality. Breast cancer has affected almost 2.3 million people worldwide in 2020, including 0.685 million fatalities (30). The new cases and deaths are presented in Table 1.

**Table 1:** New cases and deaths for different types of cancer worldwide. (Data have been acquired from Global Cancer Statistics 2020).

**Cancer type**	**New cases (% of all sites)**		**Death (% of all sites)**	
	**Number**	**%age**	**Number**	**%age**
Gastrointestinal	3,573,928	18.5	2,228,749	22.4
Leukemia	474,519	2.5	311,594	3.1
Melanoma of skin	1,198,073	6.2	63,731	0.6
Brain/Nervous system	308,102	1.0	99,840	1.0
Pulmonary cancer	2,707,406	14.7	2,019,937	20.3
Genitourinary	4,017,064	21	1,548,189	15.6
Liver cancer	1,401,450	7.3	1,296,183	13
Mouth/Oral cavity cancer	431,296	2.3	167,235	2.0
Breast cancer	2,261,419	11.7	684,996	6.9
Multiple myeloma	176,404	0.9	117,077	1.2
Mesothelioma	30,870	0.2	26,278	0.3
Hodgkin lymphoma (HL)	83,087	0.4	23,376	0.2
Mesothelioma	30,870	0.2	26,278	0.3
Kaposi sarcoma	34,270	0.2	15,086	0.2
Others	2,564,031		1,329,584	
Sum of all sites	19,292,789		9,958,133	

### Cancer Situation in Pakistan

International Agency for Research on Cancer (IARC) has reported in Pakistan that the proportion of newly diagnosed cancers is 0.18 million, the number of cancer fatalities is 0.11 million, and the number of prevalent cases (5 year) is 0.32 million (31). In Asia, Pakistan regionally represents the most significant breast cancer rate (32). Breast cancer has grown increasingly frequent in Pakistan, with one out of nine women now having a lifetime risk of the disease (33). Pakistan has one of the highest breast cancer mortality rates globally (34). Lips and mouth cancer is the 2nd most frequent malignancy in Pakistan and the top among males when both genders are included (15.9%) (35). The increased usage of smokeless tobacco, such as areca nut, can increase the burden of severe lung tumors (SLT) (36). SLT is a collection of over 30 low and high-toxic substances (37). The list of cancers, number of deaths, and new cases in Pakistan in 2020 are presented in Table 2.

**Table 2:** New cancer cases and death for different types of cancer in Pakistan (Data have been acquired from Global Cancer Statistics 2020).

**Cancer type**	**New cases (% of all sites)**		**Death (% of all sites)**	
	**Number**	**%age**	**Number**	**%age**
Gastrointestinal	23,220	14.87	21,077	18.08
Leukemia	8,305	4.7l	6,261	5.3
Melanoma of skin	502	0.28	290	0.25
Brain/Nervous system	4,770	2.7	3,934	3.4
Pulmonary tract cancer	19,008	10.61	14,488	12.31
Genitourinary	25,241	11.35	11,026	9.46
Liver Cancer and Gallbladder	8,372	4.7	7,739	6.6
Mouth/Oral cavity cancer	20,620	10.01	11,761	10.07
Breast cancer	25,928	14.5	13,725	11.7
Multiple myeloma	1,978	1.1	1,726	1.5
Mesothelioma	41	0.02	34	0.03
Non and Hodgkin lymphoma (NHL)	8,305	4.63	4,550	4.36
Mesothelioma	41	0.02	34	0.03
Kaposi sarcoma	77	0.04	51	0.04
Others	31,980	–	20,453	
Sum of all sites	178,388	–	117,149	–

## Potential Risk Factors of Cancer in Pakistan

### Diet and Nutrition

Malnutrition was common among advanced cancer patients (38). The nutritional deficiency was linked to poor clinical outcomes (38). According to numerous studies, poor nutrition is prevalent among cancer patients, with rates ranging from 31 to 97% (39–42). Malnutrition can result in impaired immunity (43), higher infection rates (44), inadequate reaction and endurance for therapy (45), higher healthcare costs (46), worse quality of life (47), and shorter survival durations (48).

#### Dietary Consumption

**Figure 2:**
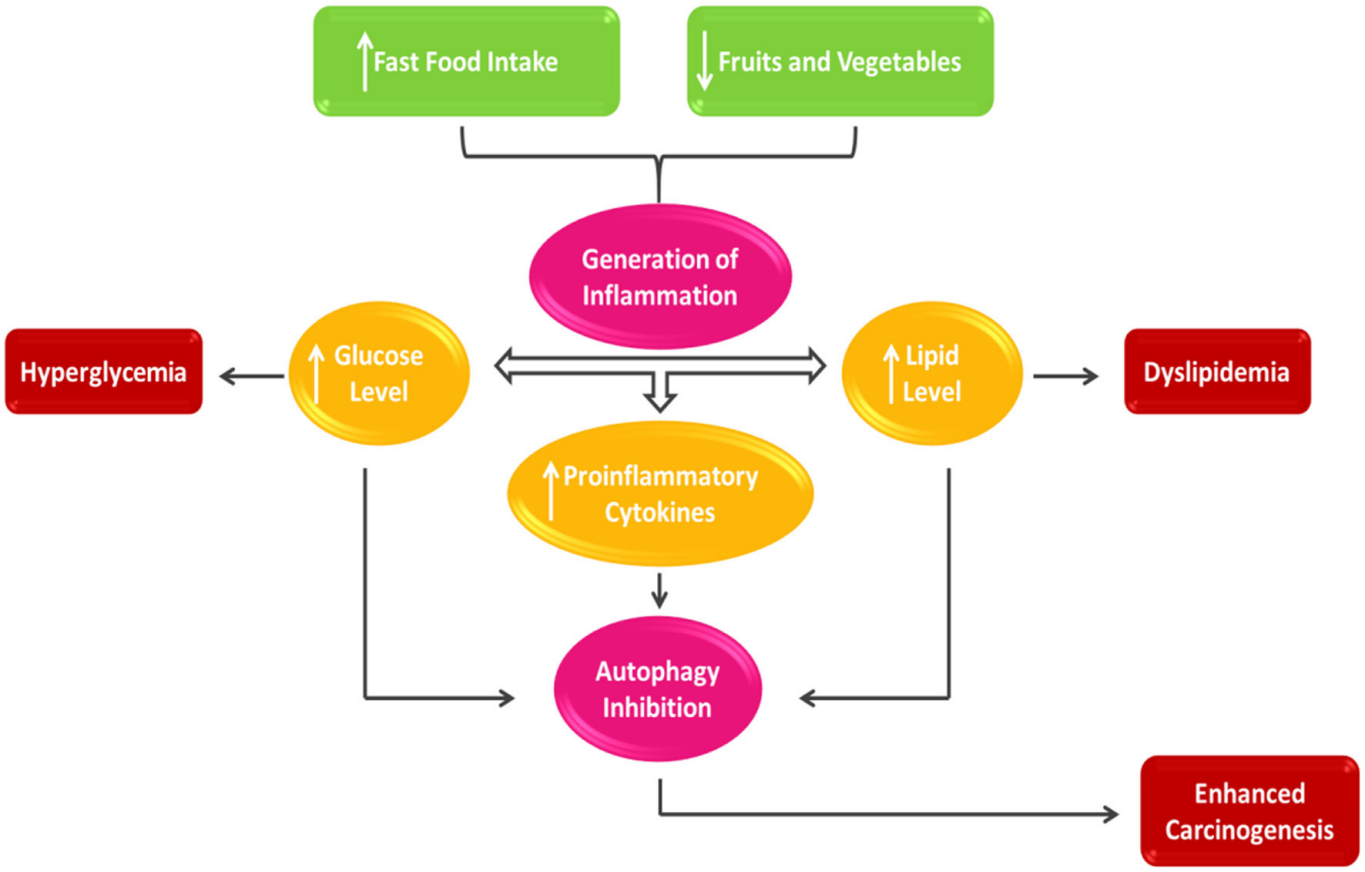
Nutrient intake and cancer prognosis.

Cereals continue to be the mainstay of the Pakistani diet, accounting for 62% of total energy (49). Pakistan has a high level of dietary patterns compared to other Asian countries (50). However, there is a lack of diet quality and fish and meat consumption (51). Fruit and fresh vegetables, which are sensitive to local seasonal availability, are similarly limited due to the country's lack of established marketing facilities. Micronutrient deficiency disorders in Pakistan are likely to be caused by fluctuations in the availability of these essential foods (52). Figure 2 shows the link between dietary consumption and cancer.

A healthy diet rich in fruits and vegetables has been related to a decreased risk of cancer. Consumption of vegetables reduces cancer risk, according to American Institute of Cancer Research (AICR) (53). As a result, it is assumed that vegetables contain chemopreventive chemicals. Isothiocyanates and myo-inositol have been the emphasis. Isothiocyanates, found as glucosinolates in green vegetables like mustard, cabbage, lettuce, radish, and cauliflower, seem plentiful (54). Beans, cereals, and nuts all contain myo-inositol (55). These compounds inhibited the enzymatic activity of 4-(N-Methyl-N-nitrosamine)-1-(3-pyridyl)-1-butanone (56, 57).

Epidemiological and experimental studies indicate that Brussels sprouts, kale, broccoli, and cabbages have anticancer properties (58). Sulforaphane, a phytochemical found in cruciferous vegetables such as broccoli, Brussels sprouts, sorghum and cabbage, has anticancer characteristics (58, 59). Cabbage has antioxidant and anti-inflammatory properties that could help prevent cancer (60). Foods that increase the bioavailable content of non-heme iron and alternative treatments for cancer patients can all benefit from fresh cabbage juice, whether made individually or combined with other vegetables like carrot and celery (61, 62) Thiocynate obtained from the hydrolysis of *Brassica oleraceae* has anticancer and antioxidant properties (60, 63). Cauliflower and cruciform vegetable intake has reduced cancer rates (64). Radish sprouts may have a more substantial chemoprotective effect against carcinogens than broccoli sprouts (65). Antimutagenic efficacy of aqueous extract of salted radish roots against *Salmonella typhimurium* TA98 and TA100 (66). Radish sprouts have a high concentration of glucoraphanin, glucosinolates that hydrolyze to produce sulforaphane, a potent inducer of phase 2 detoxification enzymes with anticancer properties (65). Due to the indoles present in turnip, it has anti-tumorigenic properties (67). Substantial antimutagenic factors and hydroxyl radical scavengers have been found in Turnip seeds (68).

Flavonoids, also present in tomatoes, have been shown to prevent carcinogenesis *in vitro*, and there is strong evidence that they can do so *in vivo* (69, 70). In considerable amounts, Tomato leaf extract includes isolated active components with anticancer activity (70). Cardioprotection, anti-inflammatory, Antimutagenic, and anti-carcinogenic qualities are only a few of the biological advantages of tomato lycopene (71). Lycopene-rich tomatoes and tomato products have decreased the risk of chronic diseases such as cancer and heart disease (71). Cancer might be prevented or delayed by delaying or inhibiting the steps leading to genetic damage or activating preventive systems (72). The large-scale (α-Tocopherol and β-carotene) cancer prevention studies and carotene and retinol effectiveness trial intervention studies found that anyone who gained early plasma or potency layers of carotenoid had a lower risk of developing lung cancer (73, 74). Those with greater pre-intervention carotene concentrations in their plasma or diets reduced cancer incidence (75). A high-carotene diet may also benefit cancer treatment (76, 77).

#### Adulteration

Pakistan is indeed an agricultural land, and farmland has always significantly influenced the nation's economy with cattle (78). Pakistan produced 42.199 million tons of milk last year, with buffaloes accounting for 62.17% of total milk output, cows 34.21%, sheep, goats, and camels 3.60%. Pakistan's milk production and delivery infrastructure are antiquated and insufficient, despite the country's favorable position among dairy-producing countries (79). The irregular private sectors, which are made up of many agents such as sellers, collectors, mediators, processors, merchants, and dairy stores, perform a specific function at a different stage of the production process and manage it (80). Pakistan's dairy industry is beset by many issues, including a scarcity of industrial milk production, milk expertise, and legal and technological resources (81).

**Figure 3:**
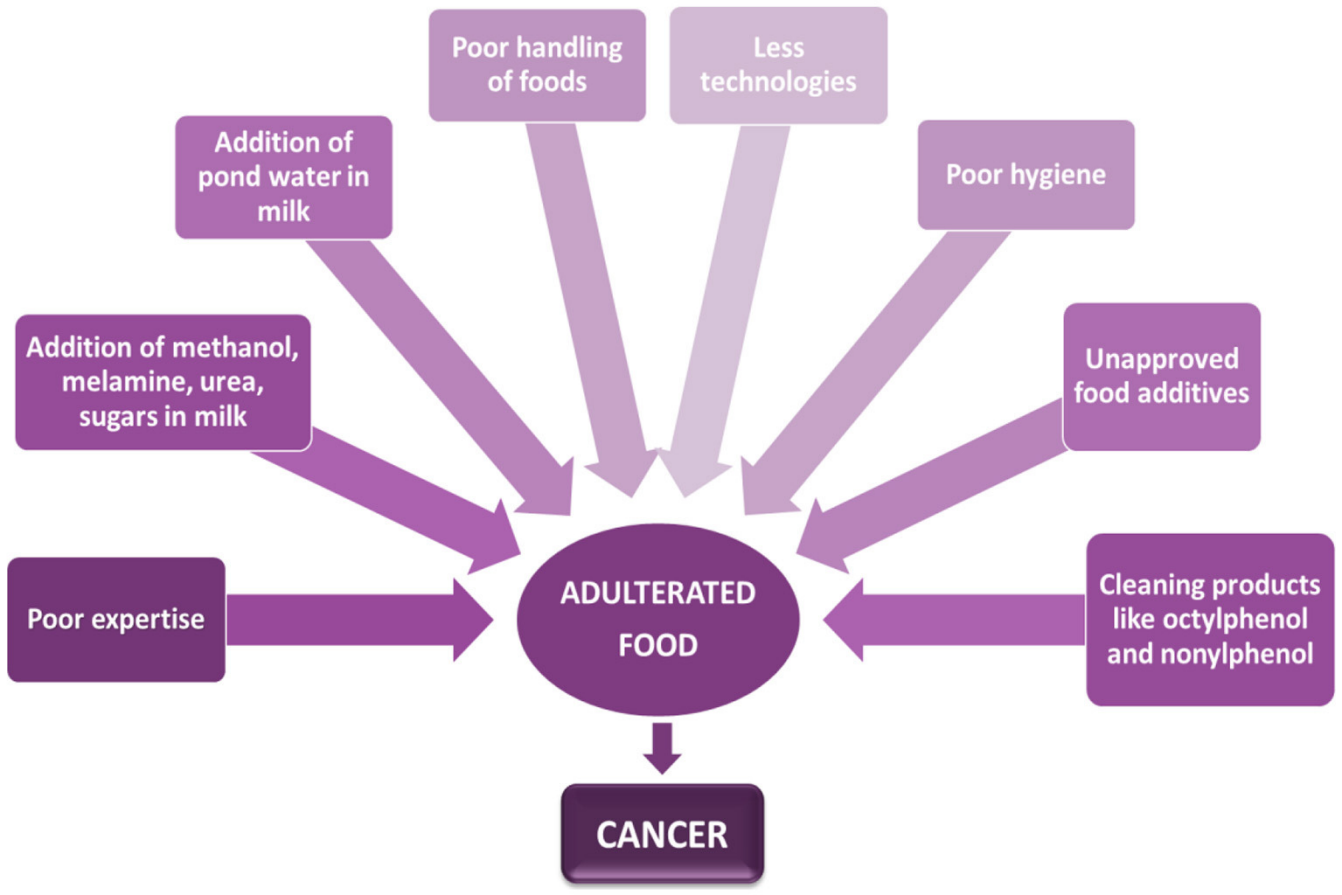
Association between contaminated food and cancer.

Moreover, its lack of decent inspection is the system's biggest overlooked flaw. At practically every stage of the marketing process, testing is just about non-existent (82). On the other hand, rising inflation and poverty levels have made most Pakistani consumers budget-conscious. As a result, open raw milk has a higher demand than pasteurized milk (83). Different factors are shown in Figure 3. Water is perhaps the most basic and transparent adulteration in milk and is applied to boost the volume of a valuable product. Still, dirty water puts people's health in danger from waterborne illnesses (84).

Owing to decreased milk output, milk yield in Pakistan is reduced by 55% throughout the summer (85). However, when milk production is abundant in contrast to the spring, price increases of up to 60% are usual. Water is blended into the whole dairy throughout the summer to enhance the milk available to meet demand (86, 87). Because pond water is an excellent nitrogen resource, some unscrupulous people add it to milk to increase the relative density. Human ingestion of these water-spoiled milk can induce gastrointestinal problems, mainly in the elderly, or pose significant health risks to children and infants who regularly eat dairy (88). Table 3 shows the many cancers induced by various foodstuffs.

**Table 3:** Linkage of certain foods to various malignancies.

**Food items**	**Type of cancer**	**References**
High intake of red meat	Colorectal cancer	(89)
High salted vegetables/food	Gastric cancer	(90)
Ultra-processed food and drinks	Chronic lymphocytic leukemia	(91)
High fat	Breast cancer	(92)
Low vitamin C, E rich foods	Skin cancer	(93)
High alcoholic beverages intake	Lung cancer	(94)
High carbs	Brain tumor	(95)
High fat	Liver cancer	(96)

To avoid milk from spoiling during delivery, especially in hot weather, it is refrigerated (the ice used may be contaminated) (97). Milk is also important because it has whey protein with many health benefits (98). Methanol, melamine, urea, and sugars can be illegally polluted in milk (97). In summers, H2O2 protects milk whenever the heat is exceptionally high. Hydrogen peroxide damage to stomach cells can cause gastritis, bowel inflammation, and severe diarrhea (99). Cleaning products used to clean milk also cause cancer (86).

#### Fast Food and Junk Intake

Fast food is food that can be prepared and served quickly (100). They're known for offering rich, delectable food at affordable costs. Nonetheless, many people nowadays, especially the young, like fast food like burgers, chicken wings, pizza, and shawarma (101). Fast food's success can be attributed to a variety of things. The changing level of living is among the essential variables. Most individuals spend extra shifts and even whole study hours to make finances meet. They won't have time to shop for supplies or prepare delectable meals. The increasing number of young, wealthy people is one factor. Most people in most countries spend a lot of money on fast food since they are primarily young (102). Children are regularly exposed to fast food advertisements on television and the Internet (103). Youngsters enjoy dishes with vivid shades and miniature gadgets, although full of fat, salty, or artificial sweeteners. Fast-food consumption has increased dramatically in Pakistan (104). Client meal preferences are impacted by several factors, including client desire to dine out (105), networking (106), globalization (107), a need for university undergraduates (108), the convenience of Pakistani households with two incomes, and a variety of other factors (109). People are eating in a new way in terms of global with the rising relative importance of snacks, burgers, pizzas, and fizzy beverages (110). As previously stated, the need for food is linked to urban culture, and development is one of the factors contributing to changing lifestyles, increasing wealth, and the independence of young people (111).

Many individuals like eating fast food regularly, even though they may be unaware of its negative health impacts. One of the ailments caused by consuming fast food is cancer (112)People in Pakistan often buy and eat fast food between 6 and 9 p.m., as per findings of a study (113). Due to its delectability, fast is consumed by single and joint households in Pakistan. The nuclear family unit is more cost-conscious than the common family unit. Fast food is popular among the general public, and many people choose to dine outdoors in their houses (114). People who were overweight were more likely to eat fast food at home (115).

As a consequence, dining out is a better choice (116). There is conflicting evidence of a relationship between food quality and prostate cancer (117). According to recent research, taking in more food, fruits, and vegetables is linked to a decreased incidence of prostate cancer (118). The low-fat, high-vegetable, and fruit-intake diet can help to reduce the risk of prostate cancer (119). In several studies, a high-fat diet, particularly red meats and dairy products, has increased cancer risk (120). In South Asia, vitamin D insufficiency is relatively frequent. According to studies, 70–97% of Pakistan's primary population is deficient in vitamin D, with vitamin D insufficiency being more prevalent in cities (121). Vitamin D deficiency is widespread among Pakistan's general population (122). Vitamin D insufficiency was detected in 95% of women with breast cancer and 77% of healthy people in research from a prestigious cancer clinic in Pakistan (123). On the other hand, Chlebowski discovered no link between vitamin D deficiencies and cancer (62, 123). In earlier studies, vitamin D levels over 50 ng/ml have been linked to a 50% decreased risk of breast cancer (124, 125).

### Gutkha and Paan

Tobacco use seems to have a long history in many regions, particularly India, Pakistan, other Asian nations, and America (126). There are around 28 carcinogenic chemical constituents in smokeless tobacco, the most common of which is nitrosamine (127). Due to a significant lack of information and awareness, most people utilize Paan and Gutkha (128).

**Figure 4:**
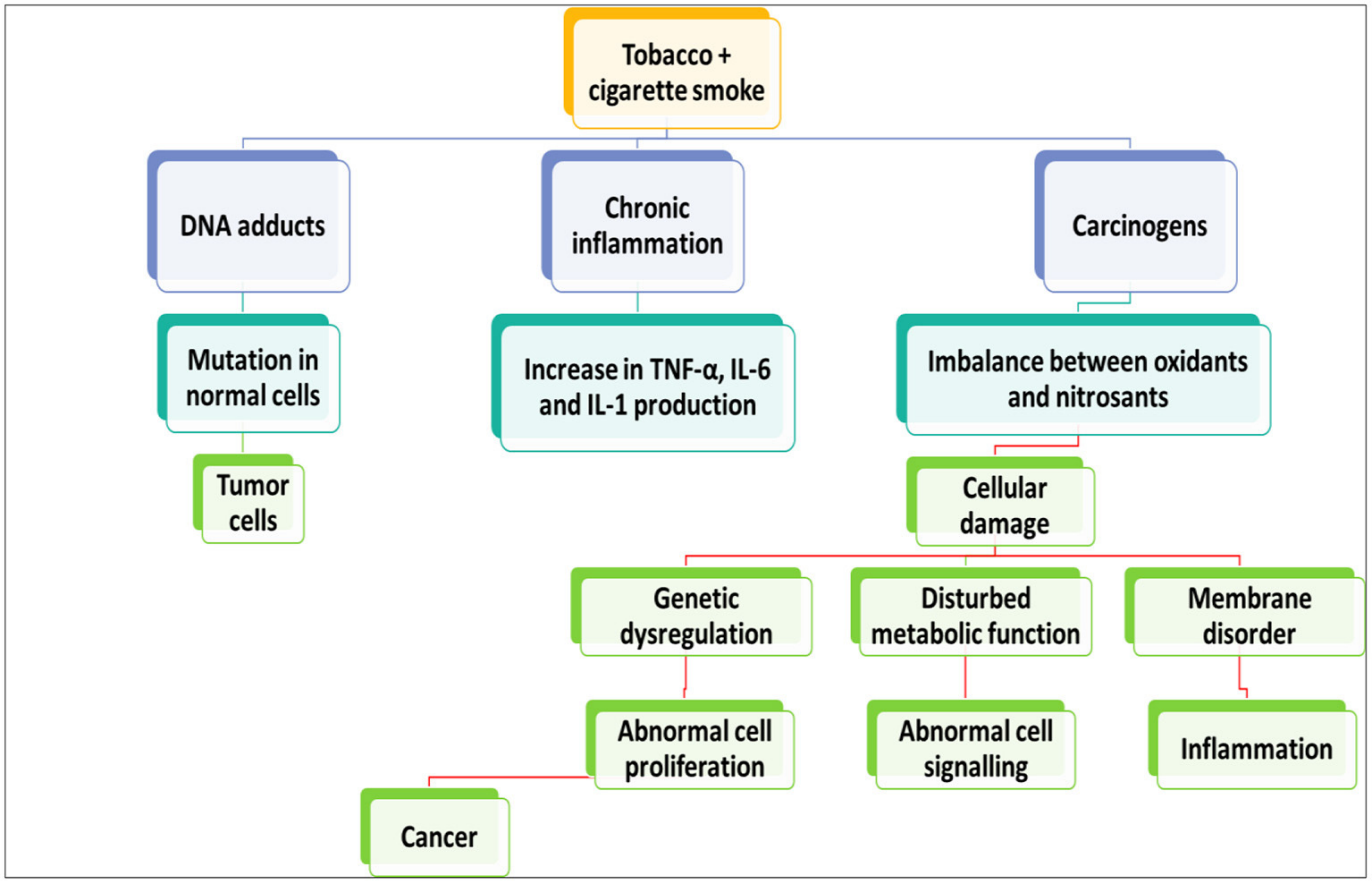
Links between the oxidative and nitrosative induced damage and progression of cancer.

Children think of Gutkha as candy because of its pleasant flavor (36). Gutkha is widely assumed to be a mouthwash (36). However, its pleasant taste and smooth texture attract bacteria, leading to tooth disease (36). In most places where Paan and Gutkha are widely used, it is difficult to control their use, and their widespread use contributes to oral cancer. Tobacco, seed, soaked lime, herbs, and geek wrapped in flake are common ingredients in paan (129). Mouth Sub Mucous Carcinoma seems to be a chronic oral condition marked by mucus buildup in the mouth, pain, and necrosis of subcutaneous soft tissue. Oral cancer is relatively frequent in Pakistan, India, China, Taiwan, Sri Lanka, Malaysia, and Indonesia (130). Gutkha intake was already proven even to have carcinogenic and chromosomal effects. In certain circumstances, drinking rather than smoking significantly impacts oral cancer (131). In Pakistan, males and females had prevalence estimates of 21.3 and 19.3%, respectively. Pakistan is the second most common country where smokeless tobacco is used (132). Tobacco products have been connected to almost 90% of mouth cancer cases, which is critical in cancer formation (133). People who inhaled smoke more than 10 times per day have been found to have a higher chance of acquiring cancer than those who did not (134). Paan is consumed by 3.3–37.0% in Pakistan and India (36). Mouth cancer is the third least frequent cancer in Pakistan and India (135), behind breast and lung cancer. Breast cancer affects more females than males (136). The use of such items is considered normal in the culture. Despite their deliciousness, Gutkha, Chaalia, paan, toombak, and naswar lead to mouth cancer (36). Numerous studies show that these goods are consumed by 20–30% of individuals and teenagers in Pakistan, India, and Nepal (137, 138). In Karachi, Pakistan, 40% of the populace chews betel nut, areca nut, and tobacco products (139). According to a study, more than 74% of pupils in Karachi, Pakistan, consume digestible items regularly (140). Paan, chaalia, gutkha, naswar, and toombak were utilized 34.3, 34.7, 46.0, and 50% of the time in Sindh, Punjab, respectively Pathan, and Mohajir districts, according to a 2006 report (141). According to a study conducted a decade ago, gutkha was consumed on a regular basis by 46% of Karachi residents (142). Another study found that 35% of persons attending a medical care facility from Karachi, Pakistan, consumed paan, gutkha, or other oral tobacco frequently (143). The paan and gutkha processes have been proposed for humans, as shown in Figure 4.

Before swallowing, paan, gutkha, and zarda are crushed, licked, or rubbed between gums and teeth. According to the World Health Organization, Nicotine mycotoxins constitute 76–91% of N-nitroso compounds (144). Paan and gutkha produce irritation of the oral mucosa, which stimulates T-cells and macrophages, resulting in the generation of prostaglandins. The pyogenic granuloma (PG) generation by buccal cavity keratinocytes is stimulated by areca nut extract, which is critical for tissue stiffness and malignancy. Tumor necrosis factor (TNF), interleukin-6, and growth factor-like have all been produced in areas of aggravation (36).

The nitrosamine in tobacco is biologically changed *via* cyp450 enzymes, which can form N-nitrosonornicotine, a carcinogenic solid that can cause DNA damage and, as a result, oral cancer (145). cytochrome P450 (CYP), Glutathione S-transferase (GST), and TNF represent cytochrome polymorphism, glutathione-s-transferase, and cancer necrosis signals.

Reduced salivation and mucus production have been observed in paan and gutkha eaters, decreasing the oral mucosa's normal microbiota and an increased risk of infection from Aspergillus (146). In laboratory animals, Paan and gutkha have been shown to cause stomach, mouth, throat, and laryngeal cancers. To mice, paan is just a melanoma agent (147). According to studies, mice given smokeless tobacco developed malignancies in their reproductive organs, ovaries, stomach, kidneys, abdomen, and lungs. As a result, smokeless tobacco is carcinogenic to the mouth and other bodily systems (148).

#### Micro-Nutrients Deficiency

A deficiency of vitamin A may cause metaplastic changes in the nasal passage (149). Both inheritance and the effects of harmful practices such as smoking and eating a diet lacking plant-based items increase the chance of secondary cancer cells in many body regions (150). The relation between micro-nutrient deficiencies with cancers has been supplemented in Table 4.

**Table 4:** Micro-nutrient deficits linked with a variety of cancers.

**Type of cancer**	**Micro-nutrients**	**Outcome**	**References**
Breast cancer	Vitamin D3	In breast cancer patients, vitamin D insufficiency is a typical occurrence.	(151)
	Selenium	In 2014, a meta-analysis identified a link between selenium blood levels and the risk of breast cancer.	(152)
	Folate, zinc, beta-carotene	In a 2014 investigation, many genetic abnormalities and/or deficits in folate, zinc, and beta-carotene were linked to triple-negative breast cancer development, especially when they were identified together.	(153)
	Iodine	Iodine is a mineral found in the thyroid and breast tissue that aids in preventing breast cancer. Low iodine levels may be considered a risk factor for breast cancer due to the high prevalence of hypothyroidism in breast cancer patients.	(154)
Prostate cancer	Zinc	A study of Nigerian prostate cancer patients identified a relationship between zinc deficit and prostate cancer and selenium and vitamin E deficiency.	(155)
	Vitamin E and trace minerals	As previously indicated, a study on Nigerian males with prostate cancer was undertaken. According to this study, prostate cancer patients exhibited significantly decreased levels of whole blood superoxide dismutase (SOD), vitamin E, serum selenium, and zinc. AS A RESULT, Vitamin E, zinc, and selenium deficiency may be risk factors for prostate cancer.	(155)
	Selenium	Increased plasma/serum selenium levels (170 ng/mL) were found to lessen the incidence of prostate cancer in a comprehensive review and meta-analysis of selenium and prostate cancer.	(156)
	Vitamin D3	Vitamin D3 25(OH) D concentrations were inversely correlated with prostate cancer risk but not vitamin D–related polymorphisms or parathyroid hormone. This suggests a relationship between low vitamin D3 blood pathology and a higher risk of prostate cancer.	(157)
Colon cancer	Folic acid	In colorectal cancer treatment, folic acid is a contentious vitamin. Even though high folate levels have been linked to a lower risk of colorectal cancer, too much folate can stimulate cancer growth.	(158)
	Selenium	In animal studies, selenium deficiency has been shown to aggravate colitis and speed tumor formation and progression in inflammatory carcinogenesis.	(159)
	Vitamin D	Many colorectal cancer patients have vitamin D3 insufficiency and deficiency.	(160)
	Fiber, low fruit and vegetable, high red and processed meat intake	Even if not a single nutrient, it has long been known that a diet poor in fruits and vegetables, fiber, and red and processed meat intake is related to the development of colorectal cancer.	(161)
Lung cancer	Selenium	Several epidemiological studies have found that persons with low selenium levels in their blood had a higher risk of lung cancer, albeit the findings are contradictory. Research done in the southeast United States showed that lower-income and black Americans were more likely to get lung cancer.	(162)
	Vitamin A	Cigarette smoking has been linked to the development of lung cancer. Cigarette smoking has been shown to lower retinoic acid levels in the lungs of rats and increase the growth of precancerous and cancerous tumors.	(163)
	Vitamin D3	Vitamin D3 deficiency is common in lung cancer patients, ranging from mild to severe.	(160)
	Zinc	Human investigations on zinc deficiency and lung cancer are few and far between. Zinc deficiency has been demonstrated to cause DNA instability and undermine its integrity in cell culture studies on human lung fibroblasts, suggesting that it may have a role in preventing DNA damage and cancer.	(164)

Vitamin C insufficiency is common among south Asians and one of the contributing causes (165). Vitamin C levels in the blood of Indians and Pakistanis living in India and Pakistan have dropped dramatically (165). The primary cause might be a lack of vitamin C rich fruits and vegetables, especially among individuals from poor socioeconomic categories (166). Patients with advanced disease receiving chemo or immunotherapy may be at a higher risk of Vitamin C depletion due to increasing demands rather than a lack of consumption (167). Women had more significant amounts of ascorbate than males, while active patients had more meaningful levels than sedentary people (168). Anemia is more common in people who have had a tumor recurrence and are in the later stage of the disease. That is, from 40% of individuals with early-stage colon cancer to almost 80% of patients with advanced cancer (169). An iron shortage can be fatal (usually related to bleeding), for those with low iron reserves or who are iron deficient somehow (170). Poverty, hunger, illiteracy, inadequate infrastructure, and a lack of policy and law are all factors that contribute to the international development association (IDA) in Pakistan, according to several indicators (171).

According to the Pakistan National Nutritional Survey Key Report (PNNSKR), 2019, Vitamin D deficiency among children under five is a severe problem in Pakistan (172). Vitamin D insufficiency was found in a substantial percentage of people (62.7%) (173). Many youngsters (13.2%) suffer from a severe deficit. The incidence is somewhat higher (63.1%) among girls than it is among boys (62.4%) (174). Vitamin D insufficiency affects the majority of women of reproductive age (WRA) (79.7%), with 54.0% having a moderate deficit and 25.7% having severe deficiency (175).Vitamin D insufficiency is more prevalent in urban areas (83.6%) than in rural areas (77.1%) (175, 176). Vitamin A deficiency in a child under the age of five affects 51.5% of children, with 12.1% suffering from severe inadequacy (175, 177). Anemia is more common in those with a tumor recurrence at a later stage of their disease (from 40% of patients with early-stage colon cancers to almost 80% of patients with advanced disease) (175, 178). Iron deficiency can be absolute (typically caused by bleeding) or functional (caused by insufficient iron reserves) (175, 179). The prevalence of the disease is somewhat higher (51.7%) in males than in girls (51.3%) (174, 175). Adolescent females in rural regions are more likely than their urban counterparts to be anemic (175, 180). Zinc Deficiency in Children under the age of five zinc deficiency affects 18.6% of the population, with comparable rates in males and girls (175). Children in rural regions had a somewhat greater likelihood (19.5%) than in large cities (17.1%) (175, 181). Some cancer types related to different nutrients are supplemented in Table 4.

## Challenges

No National Cancer Registry (NCR) in Pakistan can give the actual magnitude of the problem to create a health policy (182). Pakistan has a scarcity of nationally representative statistics. There are no long-term cancer patient data available. No comprehensive cancer control plan exists (182–184). There are currently no systematic cancer prevention and control health education efforts (183). The only national initiative was the National Program for Family Planning and Primary Health Care Pakistan (NPFPPH), which created 100 television advertisements to promote awareness of cancer's early warning signals (185). This, however, is not a long-term activity. In a few institutions, government agencies and activist groups have produced patient education materials (186); nevertheless, these activities have had limited impact. At the national, provincial, and municipal levels, a network of organizations is lacking. Low-cost public health methods to promote palliative care are lacking, particularly in low-resource areas. Due to a shortage of government money, planning was halted. Policymakers, the health sector, and other government institutions engaged in cancer care and treatment are less enthused. Pakistan lacks adequate policies and professionals for medical nutrition therapy (187). Vertical service delivery arrangements and poor performance accountability within the government are causing less efficiency and quality concerns in the health system. The private sector also duplicates services, primarily unregulated for quality treatment and cost. Pakistan's present health infrastructure are scattered and unidirectional (188). Current healthcare technology has not progressed (189). The current systems for determining the suitability of supplies, diagnostics, medications, and laboratory reagents are not based on evidence (189). There are concerns with medicine quality, pricing, and recommendations (189). The price of medicines is controversial between regulators and the pharmaceutical industry (190).

## Programs and Policies in Pakistan

### Cancer Treatment Program in Pakistan

The Pakistan Atomic Energy Commission (PAEC) has played an essential role in the health sector (191). PAEC now offers over 13 Nuclear Medicine and Oncology Centers equipped with Health and Nutrition having excellent facilities and offers to continue integrated programs to identify various malignancies and associated ailments (192). The primary disciplines accessible and in use in PAEC nuclear medical institutes are nuclear medicine, clinical oncology, surgical oncology, clinical labs, radiology, medical physics, and bioengineering (193). In addition to directing the operations of essential disciplines at several PAEC nuclear medical facilities, the Directorate General of Medical Sciences, PAEC Headquarter, is working on a Human Resource Development Program (HRDP). This would ensure that qualified and competent people in every aspect of cancer diagnosis and treatment are available (193).

### Cancer Care Hospital and Research Centre (CCHRC) Pakistan

Its purpose is to provide high-quality, all-inclusive cancer treatment at no cost while addressing their physical, social, economic, and spiritual requirements (194). They also don't turn away any patients, accepting around 20,000 each year. This hospital was created due to a lack of chemotherapy facilities in Punjab 26/110, Baluchistan 1/12, Sindh 21/4, and KPK 14/35 (195). Chemotherapy takes 4–6 months, just like it did at SKMCH&RC.

### Children Cancer Foundation Pakistan Trust (CCFPT)

This is intended for children only. This trust's mission is to offer cancer screening and treatment to all children, regardless of their financial situation. CCFPT was established years back with a strong direction and commitment to building a Children's Cancer Hospital (CCH) in Pakistan. Every child may receive quality cancer treatment and raise public awareness about childhood cancer (196).

### National Cancer Data Base Pakistan (NCDBP)

In 2010, the NCDBP was established. The Pakistan Society of Clinical Oncology (PSCO) and its associated centers also work together (197). A cancer registry must be established to prevent and manage cancer. We now need an NCR that can offer information on the actual scope of the problem to establish health policy. The Ministry of National Health Services Regulations and Coordination (NHSRC) has tasked the Pakistan Health Research Council (PHRC) with establishing a cancer registry by affiliating all of the country's leading public and private institutions. Eight hospitals (Jinnah Postgraduate Medical Center, Karachi, Civil Hospital, Karachi, National Institute of Child Health, Karachi, Nishtar Hospital, Multan, Allied Hospital Faisalabad, Bolan Hospital, Quetta, Khyber Teaching Hospital, Peshawar, and Armed Forces Institute of Pathology, Rawalpindi) have been sending data to the Human Rights Commission Pakistan (HRCP) every quarter since May 2015 (198).

### Pakistan Cancer Care Welfare Society (PCCWS)

This society's main objective is to provide financial assistance to the patient because it's challenging for someone diagnosed with cancer to manage expenses. However, Government assistance is needed too. Its mission is to help cancer patients medically, physically, socially, economically, and psychologically.

The PCCWS is a non-profit public welfare organization based in Pakistan to promote cancer awareness. PCCWS started in 2006 and presently has over 200 members and is currently striving to improve cancer awareness in lower Punjab (199). PCCWS follows the American Society of Cancer's monthly theme-based calendar system and organizes lectures, seminars, presentations, and campaigns throughout the lower Punjab, both rural and urban. PCCWS makes cancer literature in Urdu accessible, comprehensive, and up-to-date for local populations (199).

### Pakistan Institute of Medical Sciences (PIMS)

The department houses Pakistan's World Health Organization (WHO) National Cancer Control Project. The Pakistan Institute of Medical Sciences treats patients from Islamabad, Khyber Pakhtunkhwa, FATA, Gilgit-Baltistan, Kashmir, and Punjab (200). In addition, PIMS provides round-the-clock service to Parliamentarians, government officials, and judicial employees (32). All components have very visible and unambiguous signposting for services provided at PIMS to assist patients and their attendants (200). All cancers are treated at the oncology department with curative purpose chemotherapy. PIMS only have eight beds in its indoor section. Despite this, it regularly has more than 14 patients since it is the only government-run clinic in the region. The daily outpatient clinic sees 40 patients every day. Outpatient chemotherapy for 10–15 patients every day (200).

With the help of Saylani Welfare Trust and the EHSAAS Program, the patient's attendants are provided with free meals three times a day (201). In the “Shelter Home,” patient's attendants are also given a place to sleep at night (201). A “Sarai” is also available to accommodate the parents of pediatric patients (201).

### Pakistan Society of Clinical Oncology (PSCO)

PSCO is a professional organization in Pakistan for cancer/oncology experts who have come together to fight cancer nationally (202). PSCO is the largest organization in the country, representing all of the experts who treat cancer patients (202). The society's mission is to promote and support the field of clinical oncology and associated sciences in cancer treatment (202). PSCO improves clinical oncology practice as a best-cost-effective cancer strategic approach in resource-constrained countries. PSCO encourages patients and the general public to learn more about cancer care by supporting prevention, screening programs, and accurate disease information. PSCO maintains relationships with other oncology societies, cancer forums, universities, patient groups, radiotherapy machine vendors, and the pharmaceutical business (202). Its main objectives are to assist and enhance the speciality of clinical oncology and related sciences engaged in cancer therapy and to serve the community using its diagnostic, therapeutic, and research capabilities (202).

### Shaukat Khanum Memorial Cancer Hospital and Research Centre (SKMCH&RC)

Thousands of underprivileged cancer patients received free comprehensive care at the clinic in 1989 (203). It is a humanitarian institute in Punjab formed by former cricketer and Prime Minister of Pakistan Imran Khan and is primarily sponsored by donations from friends and well-wishers (203). It aims to serve as a model organization for improving the welfare of cancer patients *via* the use of current preventive and palliative therapy techniques for all cancer patients, regardless of their ability to pay, health care professionals and public education, and cancer research (203).

### Surgical Oncology Society Pakistan (SOS-PK)

SOS-PK is a professional society promoting cancer patient treatment in Pakistan by enabling interaction and lifelong learning. Pakistan is subjected to a substantial illness load (204). The SOS-PK is Pakistan's leading national cancer surgeons' representative organization. It is a member of the ESSO (European Society of Surgical Oncology) and the Society of Surgical Oncology and USA's Global Cancer Surgery Leadership Forum Initiative (204).

The goals of SOS-PK (founded in Lahore in 2008) are to promote the advancement of cancer surgery education and training by improving communications among surgeons with a primary interest in the subject (204). SOS-PK accomplishes this by hosting conferences and symposia across the country to advocate for the highest possible quality of cancer care for patients, highlighting the necessity of a multi-sectoral approach and campaigning for the establishment of comprehensive cancer hospitals that provide all treatment under one roof and by raising public awareness, it is expected to encourage cancer prevention and early detection among the general population, health administrators, and healthcare providers (204).

### Food and Nutrition

One of the most critical determinants of human resource quality is nutritional adequacy. Cancer remains a serious public health problem despite significant advancements in technology in the health and other medical industries (205). The National Action Plan for Non-Communicable Disease Prevention, Control, and Health Promotion in Pakistan (NAP-NCD) combines cancer prevention and control with a comprehensive Non-communicable Diseases (NCD) prevention framework that includes cross-cutting risks such as tobacco and food and physical activity (206). By marking cancer days and world cancer day, the PHRC hopes to improve public awareness of six common diseases of body parts (oral, lung, and liver, breast, cervical, and blood/bone marrow; schedule attached). An education pamphlet on the risk factors and prevention of common cancers has been developed in English and Urdu to easily comprehend common cancers in Pakistan. A deficiency of macronutrients and micronutrients, such as iron (207), vitamin A (208), vitamin C (209), and vitamin D (210), causes cancer (207). The major causes of such deficiencies are insufficient bioavailability and insufficient food intake. A balanced diet ensures sufficient nutrients for a healthy life (211). Malnutrition affects millions worldwide due to inadequate food intake and disease (212). Pakistan is attempting to address health-related concerns by implementing various food and nutrition initiatives.

#### Food and Nutrition Society Gilgit Baltistan (FNSGB)

FNSGB is a platform for giving people awareness about the basic needs of nutrition and healthcare. Public health issues related to food, nutrition, and health-related disorders are the major handling areas of this society (59). It gives policy and future planning for food and nutrition programs and dietary guidelines for the population on the local level (98). Delivering different campaigns having information about the cancer risk factor and their relation to the dietary patterns and food is the primary core field of this society (6, 62, 98).

#### Strategies to Avoid Cancer

Anti-smoking regulations in public places have only recently gained traction; even then, they are not being adequately enforced (213). To manage this problem, adequate measures such as anti-smoking education must be used. Advertisements have been discovered to play a substantial role in promoting smoking (214, 215). In Pakistan, regulations prohibiting such advertising techniques were enacted. In Pakistan, two principal regulations oversee tobacco control (216). Using the powers conferred by the two pieces of law. The first necessary regulation is the cigarettes (printing of warning) ordinance of 1979 (Ordinance No. LXXIII of 1979) (217). This essentially requires health warnings to be included on cigarette product packaging. Statutory regulatory orders established the regulations for publishing warnings (SROs) 86(KE)/2009 (218). The initial warning wording and graphics are established by SRO 87(E)/2009 (219). The second necessary regulation is the Ban of Smoking in Enclosed Places and Protection of Non-smokers Health Law, approved in 2002 (Ordinance No. LXXIV of 2002) (220). It controls a variety of facets of tobacco control, including public smoking prohibitions, sales to minors, cigarette advertisements, marketing, and finance. The Committee on Tobacco Advertisement Guidelines was formed by SRO 655(I)/2003 in tobacco advertising, marketing, and finance (221). Despite predictions that increasing the price of cigarettes by 10% will result in a 4.8% immediate drop and a long-term reduction of 11.7% in cigarette smoking, business economics are proving to be a pivotal hurdle to tobacco control (97). SRO 2019 will display a 60% pictorial warning label on cigarette boxes.

## Conclusion

This review focuses on cancer and its prevalence across the globe, as well as in Pakistan. Cancer is one of the leading causes of mortality around the globe. In 2025, an estimated 19.3 million more cancer cases [18.1 million excluding non-melanoma skin cancer (NMSC) and basal carcinoma] and 10 million additional cancer deaths (9.9 million excluding non-melanoma skin cancers and squamous cell carcinoma) will have been reported globally. Several cancers are also becoming more common in Pakistan. Pakistan's population is 220.9 million. According to GLOBACAN 2020, In Pakistan, there have been 178,388 new cancer cases, 117,149 cancer deaths, and 329,547 overall cancers cases found in five years. Cancer may be caused by poor nutrition, and harmful behaviors such as eating more junk food, adulterating infectious substances, gutkha, and paan. Pakistan is nutritionally deficient in vitamin D, vitamin A, zinc, and iron, according to the national nutrition survey report of 2018 (175). These nutritional deficiencies are the contributing causes of cancer. The use of gutkha, paan, fast foods and adulteration of foods are common culprits for cancer. When it comes to cancer therapy, Pakistan lacks proper policies and strategies. Different governmental and non-governmental organizations are working which are nonetheless making a substantial contribution to the health sector by applying nuclear and other cutting edge technology for cancer diagnosis and treatment. The national action plan also works on cancer prevention measures for NAP-NCD and other groups.

This is a difficult aim for Pakistan to achieve, and it will need dedication from all levels of society. Many characteristics connected to cancer burden discussed here assist to highlight features of cancer epidemiology that may be used to drive intervention programs and promote cancer determinants and outcomes research. Current data on cancer burden will be required for the establishment of national NCD action and cancer control plans, and cancer control measures must be prioritized based on local requirements. Annual updates on the cancer burden will be published in response to this demand.

## Author Contributions

AAl, MM, NA, SR, and AAh: wrote the original article. RA, HQ, RS, MM, SK, and WK: reviewed and edited the manuscript. LA and NA: supervised the manuscript. All authors contributed to the article and approved the submitted version.

## Funding

This work was supported by the Hunan Provincial Key Research and Development Program, China under grant [2018SK2065].

## Conflict of Interest

The authors declare that the research was conducted in the absence of any commercial or financial relationships that could be construed as a potential conflict of interest.

## Publisher's Note

All claims expressed in this article are solely those of the authors and do not necessarily represent those of their affiliated organizations, or those of the publisher, the editors and the reviewers. Any product that may be evaluated in this article, or claim that may be made by its manufacturer, is not guaranteed or endorsed by the publisher.

## Abbreviations

Brca1: breast cancer gene-1
Brca2: breast cancer gene-2
SKMCH&RC: Shaukat Khanum Memorial Cancer Hospital and Research Center
NHL: Non-Hodgkin lymphoma
HL: Hodgkin lymphoma
TNM: tumor nodes and metastasis
WCRF: World Cancer Research Fund
SLT: severe lung tumors
IDA: International Development Association
PNNSKR: Pakistan National Nutritional Survey Key Report
NCR: National Cancer Registry
NPFPPH: National Program for Family Planning and Primary Health Care
PAEC: Pakistan Atomic Energy Commission
HRDP: Human Resource Development Program
CCHRC: Cancer care hospital and research Centre Pakistan
CCFPT: Children Cancer Foundation Pakistan Trust
CCH: Children Cancer Hospital, Pakistan
NCDBP: National Cancer Data Base Pakistan
PSCO: Pakistan Society of Clinical Oncology
NHSRC: National Health Services Regulations and Coordination
PHRC: Pakistan Health Research Council
HRCP: Human Rights Commission Pakistan
PCCWS: Pakistan Cancer Care Welfare Society
PIMS: Pakistan Institute of Medical Sciences
SOS-PK: Surgical Oncology Society of Pakistan
NAP-NCD: National Action Plan for Non-Communicable Disease Prevention, Control and Health Promotion in Pakistan
NCD: non-communicable diseases
SROs: statutory regulatory orders
NMSC: non-melanoma skin cancer
GLOBACAN: global cancer incidence, mortality and prevalence
IARC: International agency for research on cancer
AICR: American Institute of cancer research
PG: pyogenic granuloma
TNF: tumor necrosis factor
CYP: cytochrome P450
GST: glutathione S-transferase
WRA: women of reproductive age.

## References

[1] Godet I Gilkes DM. BRCA1 and BRCA2 mutations and treatment strategies for breast cancer. Integr Cancer Sci Ther. (2017) 4:1–7. 10.15761/ICST.1000228 28706734 PMC5505673

[2] Lowenfels AB Maisonneuve P. Epidemiology and risk factors for pancreatic cancer. Best Pract Res Clin Gastroenterol. (2006) 20:197–209. 10.1016/j.bpg.2005.10.001 16549324

[3] Kampa M Castanas E. Human health effects of air pollution. Environ. Pollut. (2008) 151:362–7. 10.1016/j.envpol.2007.06.012 17646040

[4] Duell EJ. Epidemiology and potential mechanisms of tobacco smoking and heavy alcohol consumption in pancreatic cancer. Mol Carcinog. (2012) 51:40–52. 10.1002/mc.20786 22162230

[5] Williams PD Piamjariyakul U Ducey K Badura J Boltz KD Olberding K. Cancer treatment, symptom monitoring, and self-care in adults: pilot study. Cancer Nurs. (2006) 29:347–55. 10.1097/00002820-200609000-00001 17006107

[6] Ali A Mughal H Ahmad N Babar Q Saeed A Khalid W. Novel therapeutic drug strategies to tackle immune-oncological challenges faced by cancer patients during COVID-19. Expert Rev Anticancer Ther. (2021) 21:1371–83. 10.1080/14737140.2021.1991317 34643141

[7] WebMD Cancer Center,. Understanding Cancer – the Basics. (2020). Available online at: https://www.webmd.com/cancer/guide/understanding-cancer-basics (accessed March 12, 2022).

[8] Elliott P Shaddick G Douglass M de Hoogh K Briggs DJ Toledano MB. Adult cancers near high-voltage overhead power lines. Epidemiology. (2013) 24:184–90. 10.1097/EDE.0b013e31827e95b9 23337237

[9] Center SKMCHaR,. Cancer Statistics. (2020). Available online at: https://shaukatkhanum.org.pk/health-care-professionals-researchers/cancer-statistics/ (accessed March 16, 2022).

[10] Turcotte LM Neglia JP Reulen RC Ronckers CM Van Leeuwen FE Morton LM. Risk, risk factors, and surveillance of subsequent malignant neoplasms in survivors of childhood cancer: a review. J Clin Oncol. (2018) 36:2145. 10.1200/JCO.2017.76.7764 29874133 PMC6075849

[11] Anis R Gaballah K. Oral cancer in the UAE: a multicenter, retrospective study. Libyan J Med. (2013) 8:21782. 10.3402/ljm.v8i0.21782 23985381 PMC3756533

[12] De Angelis R Francisci S Baili P Marchesi F Roazzi P Belot A. The EUROCARE-4 database on cancer survival in Europe: data standardisation, quality control and methods of statistical analysis. Eur J Cancer. (2009) 45:909–30. 10.1016/j.ejca.2008.11.003 19128955

[13] Hochberg J Waxman IM Kelly KM Morris E Cairo MS. Adolescent non-Hodgkin lymphoma and Hodgkin lymphoma: state of the science. Br J Haematol. (2009) 144:24–40. 10.1111/j.1365-2141.2008.07393.x 19087093

[14] Torre LA Siegel RL Ward EM Jemal A. Global cancer incidence and mortality rates and trends-an update. Cancer Epidemiol Prevent Biomark. (2016) 25:16–27. 10.1158/1055-9965.EPI-15-0578 26667886

[15] Barbeiro S Atalaia-Martins C Marcos P Gonçalves C Cotrim I Vasconcelos H. case series of anal carcinoma misdiagnosed as idiopathic chronic anal fissure. GE-Portug J Gastroenterol. (2017) 24:227–31. 10.1159/000452869 29255757 PMC5729951

[16] American Cancer Society,. Cancer Staging. (2021). Available online at: https://www.cancer.org/treatment/understanding-your-diagnosis/staging.html (accessed March 17, 2022).

[17] Feitelson MA Arzumanyan A Kulathinal RJ Blain SW Holcombe RF Mahajna J. Sustained proliferation in cancer: mechanisms and novel therapeutic targets. Semin Cancer Biol. (2015) 35(Suppl.):S25–54. 10.1016/j.semcancer.2015.02.006 25892662 PMC4898971

[18] Labi V Erlacher M. How cell death shapes cancer. Cell Death Dis. (2015) 6:e1675.e. 10.1038/cddis.2015.20 25741600 PMC4385913

[19] Egelund R Petersen H. The plasminogen activation system in tumor growth, invasion, and metastasis. Cell Mol Life Sci. (2000) 57:25–40. 10.1007/s000180050497 10949579 PMC11146824

[20] Marx RE Ehler WJ Tayapongsak P Pierce LW. Relationship of oxygen dose to angiogenesis induction in irradiated tissue. Am J Surg. (1990) 160:519–24. 10.1016/S0002-9610(05)81019-0 2240387

[21] Gentry M. World Cancer Research Fund International (WCRF). Impact. (2017) 2017:32–3. 10.21820/23987073.2017.4.32

[22] World Cancer Research Fund International. Research Findings. (2021). Available online at: https://www.wcrf.org/research-we-fund/research-findings/ (accessed March 22, 2022).

[23] Hartwell LH Unger MW. Unequal division in Saccharomyces cerevisiae and its implications for the control of cell division. J Cell Biol. (1977) 75:422–35. 10.1083/jcb.75.2.422 400873 PMC2109951

[24] Sumner AT. Chromosomes: Organization and Function. Malden, MA: John Wiley & Sons (2008).

[25] Kastan MB Bartek J. Cell-cycle checkpoints and cancer. Nature. (2004) 432:316–23. 10.1038/nature03097 15549093

[26] Valko M Rhodes C Moncol J Izakovic M Mazur M. Free radicals, metals and antioxidants in oxidative stress-induced cancer. Chem Biol Interact. (2006) 160:1–40. 10.1016/j.cbi.2005.12.009 16430879

[27] Loeb LA Harris CC. Advances in chemical carcinogenesis: a historical review and prospective. Cancer Res. (2008) 68:6863–72. 10.1158/0008-5472.CAN-08-2852 18757397 PMC2583449

[28] Sung H Ferlay J Siegel RL Laversanne M Soerjomataram I Jemal A. Global cancer statistics 2020: GLOBOCAN estimates of incidence and mortality worldwide for 36 cancers in 185 countries. CA Cancer J Clin. (2021) 71:209–49. 10.3322/caac.21660 33538338

[29] Ferlay J Colombet M Soerjomataram I Parkin DM Piñeros M Znaor A. Cancer statistics for the year 2020: an overview. Int J Cancer. (2021) 149:778–89. 10.1002/ijc.33588 33818764

[30] Lei S Zheng R Zhang S Wang S Chen R Sun K. Global patterns of breast cancer incidence and mortality: a population-based cancer registry data analysis from 2000 to 2020. Cancer Commun. (2021) 41:1183–94. 10.1002/cac2.12207 34399040 PMC8626596

[31] Hussain I Majeed A Rasool MF Hussain M Imran I Ullah M. Knowledge, attitude, preventive practices and perceived barriers to screening about colorectal cancer among university students of newly merged district, Kpk, Pakistan-A cross-sectional study. J Oncol Pharm Pract. (2021) 27:359–67. 10.1177/1078155220922598 32390538

[32] Rashid A Aqeel M Malik B Salim S. The prevalence of psychiatric disorders in breast cancer patients; a cross-sectional study of breast cancer patients experience in Pakistan. Nature. (2021) 1:1–7. 10.47391/NNJP.01

[33] Sohail S Alam SN. Breast cancer in Pakistan-awareness and early detection (2007). Available online at: https://ecommons.aku.edu/cgi/viewcontent.cgi?article=1449&context=pakistan_fhs_mc_radiol 18182132

[34] Sarwar MR Saqib A. Cancer prevalence, incidence and mortality rates in Pakistan in 2012. Cogent Med. (2017) 4:1288773. 10.1080/2331205X.2017.1288773

[35] Anwar N Pervez S Chundriger Q Awan S Moatter T Ali TS. Oral cancer: Clinicopathological features and associated risk factors in a high risk population presenting to a major tertiary care center in Pakistan. PLoS ONE. (2020) 15:e0236359. 10.1371/journal.pone.0236359 32760151 PMC7410283

[36] Niaz K Maqbool F Khan F Bahadar H Hassan FI Abdollahi M. Smokeless tobacco (paan and gutkha) consumption, prevalence, and contribution to oral cancer. Epidemiol Health. (2017) 39:e2017009. 10.4178/epih.e2017009 28292008 PMC5543298

[37] Siddiqi K Shah S Abbas SM Vidyasagaran A Jawad M Dogar O. Global burden of disease due to smokeless tobacco consumption in adults: analysis of data from 113 countries. BMC Med. (2015) 13:194. 10.1186/s12916-015-0424-2 26278072 PMC4538761

[38] Aktas A Walsh D Galang M O'Donoghue N Rybicki L Hullihen B. Underrecognition of malnutrition in advanced cancer: the role of the dietitian and clinical practice variations. Am J Hosp Palliat Med. (2017) 34:547–55. 10.1177/1049909116639969 27069100

[39] Bossi P Delrio P Mascheroni A Zanetti M. The spectrum of malnutrition/cachexia/sarcopenia in oncology according to different cancer types and settings: a narrative review. Nutrients. (2021) 13:1980. 10.3390/nu13061980 34207529 PMC8226689

[40] Ravasco P. Nutrition in cancer patients. J Clin Med. (2019) 8:1211. 10.3390/jcm8081211 31416154 PMC6723589

[41] Planas M Álvarez-Hernández J León-Sanz M Celaya-Pérez S Araujo K García de. Lorenzo A. Prevalence of hospital malnutrition in cancer patients: a sub-analysis of the PREDyCES® study. Support Care Cancer. (2016) 24:429–35. 10.1007/s00520-015-2813-7 26099900

[42] Capra S Ferguson M Ried K. Cancer: impact of nutrition intervention outcome-nutrition issues for patients. Nutrition. (2001) 17:769–72. 10.1016/S0899-9007(01)00632-3 11527676

[43] Bourke CD Berkley JA Prendergast AJ. Immune dysfunction as a cause and consequence of malnutrition. Trends Immunol. (2016) 37:386–98. 10.1016/j.it.2016.04.003 27237815 PMC4889773

[44] Barker LA Gout BS Crowe TC. Hospital malnutrition: prevalence, identification and impact on patients and the healthcare system. Int J Environ Res Public Health. (2011) 8:514–27. 10.3390/ijerph8020514 21556200 PMC3084475

[45] Dasarathy S Merli M. Sarcopenia from mechanism to diagnosis and treatment in liver disease. J Hepatol. (2016) 65:1232–44. 10.1016/j.jhep.2016.07.040 27515775 PMC5116259

[46] Freijer K Tan SS Koopmanschap MA Meijers JM Halfens RJ Nuijten MJ. The economic costs of disease related malnutrition. Clin Nutr. (2013) 32:136–41. 10.1016/j.clnu.2012.06.009 22789931

[47] Rasheed S Woods RT. Malnutrition and quality of life in older people: a systematic review and meta-analysis. Ageing Res Rev. (2013) 12:561–6. 10.1016/j.arr.2012.11.003 23228882

[48] Lummaa V Clutton-Brock T. Early development, survival and reproduction in humans. Trends Ecol Evol. (2002) 17:141–7. 10.1016/S0169-5347(01)02414-4

[49] Shiferaw B Smale M Braun H-J Duveiller E Reynolds M Muricho G. Crops that feed the world 10. Past successes and future challenges to the role played by wheat in global food security. Food Secur. (2013) 5:291–317. 10.1007/s12571-013-0263-y

[50] Holmboe-Ottesen G Wandel M. Changes in dietary habits after migration and consequences for health: a focus on South Asians in Europe. Food Nutr Res. (2012) 56:18891. 10.3402/fnr.v56i0.18891 23139649 PMC3492807

[51] Ali A Shaukat H Ahmed M Bostani A Hussain S. Relation of electrical stimulation to meat standard. Vet Sci. (2021) 7:42–51. 10.17582/journal.vsrr/2021.7.1.42.51

[52] Mellin-Olsen T Wandel M. Changes in food habits among Pakistani immigrant women in Oslo, Norway. Ethn Health. (2005) 10:311–39. 10.1080/13557850500145238 16191730

[53] World Cancer Research Fund/American Institute for Cancer Research (WCRF/AICR). Food, Nutrition, Physical Activity, and the Prevention of Cancer: A Global Perspective. Washington, DC: WCRF/AICR (2007).

[54] Fahey JW Zalcmann AT Talalay P. The chemical diversity and distribution of glucosinolates and isothiocyanates among plants. Phytochemistry. (2001) 56:5–51. 10.1016/S0031-9422(00)00316-2 11198818

[55] Clements Jr RS Darnell B. Myo-inositol content of common foods: development of a high-myo-inositol diet. Am J Clin Nutr. (1980) 33:1954–67. 10.1093/ajcn/33.9.1954 7416064

[56] Hecht SS. Inhibition of carcinogenesis by isothiocyanates. Drug Metab Rev. (2000) 32:395–411. 10.1081/DMR-100102342 11139137

[57] Hecht SS Kenney PM Wang M Upadhyaya P. Dose-response study of myo-inositol as an inhibitor of lung tumorigenesis induced in A/J mice by benzo [a] pyrene and 4-(methylnitrosamino)-1-(3-pyridyl)-1-butanone. Cancer Lett. (2001) 167:1–6. 10.1016/S0304-3835(01)00454-2 11323092

[58] Maruthanila V Poornima J Mirunalini S. Attenuation of carcinogenesis and the mechanism underlying by the influence of indole-3-carbinol and its metabolite 3, 3′-diindolylmethane: a therapeutic marvel. Adv Pharmacol Sci. (2014) 2014:832161. 10.1155/2014/832161 24982671 PMC4060499

[59] Khalid W Ali A Arshad MS Afzal F Akram R Siddeeg A. Nutrients and bioactive compounds of *Sorghum bicolor* L. used to prepare functional foods: a review on the efficacy against different chronic disorders. Int J Food Proper. (2022) 25:1045–62. 10.1080/10942912.2022.2071293

[60] Rokayya S Li C-J Zhao Y Li Y Sun C-H. Cabbage (*Brassica oleracea* L. var capitata) phytochemicals with antioxidant and anti-inflammatory potential Asian Pacific. J Cancer Prevent. (2013) 14:6657–62. 10.7314/APJCP.2013.14.11.6657 24377584

[61] Craig WJ. Nutrition concerns and health effects of vegetarian diets. Nutr Clin Pract. (2010) 25:613–20. 10.1177/0884533610385707 21139125

[62] Babar Q Ali A Saeed A Tahir MF. Novel treatment strategy against COVID-19 through anti-inflammatory, antioxidant and immunostimulatory properties of the b vitamin complex. In: B-Complex Vitamins: Sources, Intakes and Novel Applications. Intechopen (2021). 10.5772/intechopen.100251

[63] Farag MA Motaal AAA. Sulforaphane composition, cytotoxic and antioxidant activity of crucifer vegetables. J Adv Res. (2010) 1:65–70. 10.1016/j.jare.2010.02.005

[64] Abbaoui B Lucas CR Riedl KM Clinton SK Mortazavi A. Cruciferous vegetables, isothiocyanates, and bladder cancer prevention. Mol Nutr Food Res. (2018) 62:1800079. 10.1002/mnfr.201800079 30079608 PMC6196731

[65] Liang H Wei Y Li R Cheng L Yuan Q Zheng F. Intensifying sulforaphane formation in broccoli sprouts by using other cruciferous sprouts additions. Food Sci Biotechnol. (2018) 27:957–62. 10.1007/s10068-018-0347-8 30263824 PMC6085252

[66] Gautam S Saxena S Kumar S. Fruits and vegetables as dietary sources of antimutagens. J Food Chem Nanotechnol. (2016) 2:97–114. 10.17756/jfcn.2016-01828110066

[67] Banerjee S Kong D Wang Z Bao B Hillman GG Sarkar FH. Attenuation of multi-targeted proliferation-linked signaling by 3, 3′-diindolylmethane (DIM): from bench to clinic. Mutat Res Rev. (2011) 728:47–66. 10.1016/j.mrrev.2011.06.001 21703360 PMC4120774

[68] Arora S Vig AP. Inhibition of DNA oxidative damage and antimutagenic activity by dichloromethane extract of *Brassica rapa* var. rapa L seeds. Indus Crops Prod. (2015) 74:585–91. 10.1016/j.indcrop.2015.05.038

[69] Ren W Qiao Z Wang H Zhu L Zhang L. Flavonoids: promising anticancer agents. Med Res Rev. (2003) 23:519–34. 10.1002/med.10033 12710022

[70] Kaur C Kapoor HC. Antioxidants in fruits and vegetables-the millennium's health. Int J Food Sci Technol. (2001) 36:703–25. 10.1046/j.1365-2621.2001.00513.x

[71] Chaudhary P Sharma A Singh B Nagpal AK. Bioactivities of phytochemicals present in tomato. J Food Sci Technol. (2018) 55:2833–49. 10.1007/s13197-018-3221-z 30065393 PMC6045986

[72] D'Archivio M Santangelo C Scazzocchio B Varì R Filesi C Masella R. Modulatory effects of polyphenols on apoptosis induction: relevance for cancer prevention. Int J Mol Sci. (2008) 9:213–28. 10.3390/ijms9030213 19325744 PMC2635670

[73] Das Gupta S Suh N. Tocopherols in cancer: an update. Mol Nutr Food Res. (2016) 60:1354–63. 10.1002/mnfr.201500847 26751721 PMC4899293

[74] Eggersdorfer M Wyss A. Carotenoids in human nutrition and health. Arch Biochem Biophys. (2018) 652:18–26. 10.1016/j.abb.2018.06.001 29885291

[75] Schmidt KM Haddad EN Sugino KY Vevang KR Peterson LA Koratkar R. Dietary and plasma carotenoids are positively associated with alpha diversity in the fecal microbiota of pregnant women. J Food Sci. (2021) 86:602–13. 10.1111/1750-3841.15586 33449409 PMC10035785

[76] Brewczyński A Jabłońska B Kentnowski M Mrowiec S Składowski K Rutkowski T. The association between carotenoids and head and neck cancer risk. Nutrients. (2022) 14:88. 10.3390/nu14010088 35010963 PMC8746385

[77] van den Brandt PA. Red meat, processed meat, and other dietary protein sources and risk of overall and cause-specific mortality in The Netherlands Cohort Study. Eur J Epidemiol. (2019) 34:351–69. 10.1007/s10654-019-00483-9 30673923 PMC6451725

[78] Saqib SE Kuwornu JK Panezia S Ali U. Factors determining subsistence farmers' access to agricultural credit in flood-prone areas of Pakistan. Kasetsart J Soc Sci. (2018) 39:262–8. 10.1016/j.kjss.2017.06.001

[79] Staal SJ Nin Pratt A Jabbar M. Dairy development for the resource poor. In: Part 3: Pakistan and India Dairy Development Case Studies FAO/PPLPI Working Paper. Rome: Food and Agriculture Organization (2008).

[80] Vorley B Lundy M MacGregor J. Business Models That Are Inclusive of Small Farmers. Agro-industries for Development. Wallingford: CABI for FAO and UNIDO (2009). p. 186–222. 10.1079/9781845935764.0186

[81] Tahir MN Riaz R Bilal M Nouman HM. Current standing and future challenges of dairying in Pakistan: a status update. In: Javed K editor. Milk Production, Processing and Marketing. London (2019). p. 1–117.

[82] Awan A Naseer M Iqbal A Ali M Iqbal R Iqbal F. A study on chemical composition and detection of chemical adulteration in tetra pack milk samples commercially available in Multan. Pakistan J Pharm Sci. (2014) 27:183–6. Available online at: http://www.pjps.pk/wp-content/uploads/pdfs/27/1/Paper-27.pdf 24374447

[83] Jalil H Rehman HU Sial MH Hussain SS. Analysis of milk production system in Peri-urban areas of Lahore (Pakistan): a case study. Pakistan Econ Soc Rev. (2009) 47:229–42. Available online at: http://www.jstor.org/stable/25825354

[84] Motta TC Hoff R Barreto F Andrade R Lorenzini D Meneghini L. Detection and confirmation of milk adulteration with cheese whey using proteomic-like sample preparation and liquid chromatography-electrospray-tandem mass spectrometry analysis. Talanta. (2014) 120:498–505. 10.1016/j.talanta.2013.11.093 24468402

[85] Javed K Afzal M Sattar A Mirza R. Environmental factors affecting milk yield in Friesian cows in Punjab, Pakistan. Pak Vet J. (2004) 24:58–61. Available online at: http://www.pvj.com.pk/pdf-files/24_2/58-61.pdf

[86] Handford CE Campbell K Elliott CT. Impacts of milk fraud on food safety and nutrition with special emphasis on developing countries. Comprehens Rev Food Sci Food Saf. (2016) 15:130–42. 10.1111/1541-4337.12181 33371582

[87] KC B Schultz B McIndoe I Rutter H Dark A Prasad K. Impacts of dairy farming systems on water quantity and quality in Brazil, Ethiopia, Nepal, New Zealand and the USA. Irrigat Drain. (2020) 69:944–55. 10.1002/ird.2486

[88] Singh P Gandhi N. Milk preservatives and adulterants: processing, regulatory and safety issues. Food Rev Int. (2015) 31:236–61. 10.1080/87559129.2014.994818

[89] Boada LD Henríquez-Hernández LA Luzardo O. The impact of red and processed meat consumption on cancer and other health outcomes: Epidemiological evidences. Food Chem Toxicol. (2016) 92:236–44. 10.1016/j.fct.2016.04.008 27106137

[90] Peleteiro B Lopes C Figueiredo C Lunet N. Salt intake and gastric cancer risk according to *Helicobacter pylori* infection, smoking, tumour site and histological type. Br J Cancer. (2011) 104:198–207. 10.1038/sj.bjc.6605993 21081930 PMC3039805

[91] Solans M Castelló A Benavente Y Marcos-Gragera R Amiano P Gracia-Lavedan E. Adherence to the Western, Prudent, and Mediterranean dietary patterns and chronic lymphocytic leukemia in the MCC-Spain study. Haematologica. (2018) 103:1881. 10.3324/haematol.2018.192526 29954942 PMC6278961

[92] Wu AH Pike MC Stram DO. Meta-analysis: dietary fat intake, serum estrogen levels, and the risk of breast cancer. J Natl Cancer Inst. (1999) 91:529–34. 10.1093/jnci/91.6.529 10088623

[93] Black HS Chan JT. Suppression of ultraviolet light-induced tumor formation by dietary antioxidants. J Invest Dermatol. (1975) 65:412–4. 10.1111/1523-1747.ep12607661 1176794

[94] Carbamate E,. Volume 96: Alcohol Consumption Ethyl Carbamate. World Health Organization International Agency for Research on Cancer (2010). p. 632–714. Available online at: https://publications.iarc.fr/Book-And-Report-Series/Iarc-Monographs-On-The-Identification-Of-Carcinogenic-Hazards-To-Humans/Alcohol-Consumption-And-Ethyl-Carbamate-2010

[95] Mukherjee P Seyfried T. Metabolic targeting of brain cancer. Nutr Metab (Lond). (2005) 2:30. 10.1186/1743-7075-2-30 16242042 PMC1276814

[96] Caldwell SH Crespo DM Kang HS Al-Osaimi AM. Obesity and hepatocellular carcinoma. Gastroenterology. (2004) 127:S97–103. 10.1053/j.gastro.2004.09.021 15508109

[97] Ahmad SA Ahmed M Qadir MA Shafiq MI Batool N Nosheen N. Quantitation and risk assessment of chemical adulterants in milk using UHPLC coupled to photodiode array and differential refractive index detectors. Food Anal Methods. (2016) 9:3367–76. 10.1007/s12161-016-0534-2

[98] Ali A Ain Q Saeed A Khalid W Ahmed M Bostani A. Bio-molecular characteristics of whey proteins with relation to inflammation. In: New Advances in the Dairy Industry. Intechopen (2021). 10.5772/intechopen.99220

[99] Patel PK Patel SK Dixit S Rathore R. Gastritis and peptic ulcer diseases in dogs: A review. Int J Curr Microbiol App Sci. (2018) 7:2475–501. 10.20546/ijcmas.2018.703.288

[100] Xiao A Yang S Iqbal Q. Factors affecting purchase intentions in generation Y: an empirical evidence from fast food industry in Malaysia. Administr Sci. (2018) 9:4. 10.3390/admsci9010004

[101] Musaiger AO. Consumption, health attitudes and perception toward fast food among Arab consumers in Kuwait: gender differences. Glob J Health Sci. (2014) 6:136. 10.5539/gjhs.v6n6p136 25363129 PMC4825490

[102] Tannock S. Youth at Work: The Unionized Fast-Food and Grocery Workplace. Philadelphia, PA: Temple University Press (2001).

[103] Harris JL Schwartz MB Brownell KD. Evaluating Fast Food Nutrition and Marketing to Youth. New Haven, CT: Yale Rudd Center for Food Policy & Obesity (2010).

[104] Baig AK Saeed M. Review of trends in fast food consumption. Eur J Econ Finan Administr Sci. (2012) 48:77–85. Available online at: https://www.researchgate.net/profile/Munazza-Saeed-3/publication/266627067_Review_of_Trends_in_Fast_Food_Consumption/links/543652160cf2dc341db2fa61/Review-of-Trends-in-Fast-Food-Consumption.pdf

[105] Blisard WN Jolliffe D. Let's Eat Out: Americans Weigh Taste, Convenience and Nutrition. US Department of Agriculture, Economic Research Service (2006).

[106] Cohen DA Babey SH. Contextual influences on eating behaviours: heuristic processing and dietary choices. Obes Rev. (2012) 13:766–79. 10.1111/j.1467-789X.2012.01001.x 22551473 PMC3667220

[107] Mead E Gittelsohn J Kratzmann M Roache C Sharma S. Impact of the changing food environment on dietary practices of an Inuit population in Arctic Canada. J Hum Nutr Dietet. (2010) 23:18–26. 10.1111/j.1365-277X.2010.01102.x 21158958

[108] James D. Factors influencing food choices, dietary intake, and nutrition-related attitudes among African Americans: application of a culturally sensitive model. Ethn Health. (2004) 9:349–67. 10.1080/1355785042000285375 15570680

[109] Harrington RJ Ottenbacher MC Kendall K. Fine-dining restaurant selection: Direct and moderating effects of customer attributes. J Foodservice Bus Res. (2011) 14:272–89. 10.1080/15378020.2011.594388

[110] Anand R. A. study of determinants impacting consumers food choice with reference to the fast food consumption in India. Soc Bus Rev. (2011) 6:176–187. 10.1108/17465681111143993

[111] Viner RM Ozer EM Denny S Marmot M Resnick M Fatusi A. Adolescence and the social determinants of health. Lancet. (2012) 379:1641–52. 10.1016/S0140-6736(12)60149-4 22538179

[112] Fuhrman J. The hidden dangers of fast and processed food. Am J Lifestyle Med. (2018) 12:375–81. 10.1177/1559827618766483 30283262 PMC6146358

[113] Yahya F Zafar R Shafiq S. Trend of fast food consumption and its effect on Pakistani society. Food Sci Qual Manage. (2013) 11:1–7. Available online at: https://core.ac.uk/download/pdf/234683616.pdf

[114] Yan Y. Of Hamburger and Social Space: Consuming. Beijing: McDonald's (2000).

[115] Boutelle KN Fulkerson JA Neumark-Sztainer D Story M French SA. Fast food for family meals: relationships with parent and adolescent food intake, home food availability and weight status. Public Health Nutr. (2007) 10:16–23. 10.1017/S136898000721794X 17212838

[116] Abraham S Martinez M Salas G Smith J. College student's perception of risk factors related to fast food consumption and their eating habits. J Nutr Hum Health. (2018) 2:18–21. 10.35841/nutrition-human-health.2.1.18-21

[117] Meng H Hu W Chen Z Shen Y. Fruit and vegetable intake and prostate cancer risk: a meta-analysis. Asia-Pac J Clin Oncol. (2014) 10:133–40. 10.1111/ajco.12067 23551391

[118] Castelló A Boldo E Amiano P Castaño-Vinyals G Aragonés N Gómez-Acebo I. Mediterranean dietary pattern is associated with low risk of aggressive prostate cancer: MCC-Spain Study. J Urol. (2018) 199:430–7. 10.1016/j.juro.2017.08.087 28842246

[119] Dieli-Conwright CM Lee K Kiwata JL. Reducing the risk of breast cancer recurrence: an evaluation of the effects and mechanisms of diet and exercise. Curr Breast Cancer Rep. (2016) 8:139–50. 10.1007/s12609-016-0218-3 27909546 PMC5112289

[120] Xu X Li J Wang X Wang S Meng S Zhu Y. Tomato consumption and prostate cancer risk: a systematic review and meta-analysis. Sci Rep. (2016) 6:1–8. 10.1038/srep37091 27841367 PMC5107915

[121] Iqbal R Khan AH. Possible causes of vitamin D deficiency (VDD) in Pakistani population residing in Pakistan. J Pak Med Assoc. (2010) 60:1–2. Available online at: https://www.jpma.org.pk/article-details/1888 20055268

[122] Khan H Ansari M Waheed U Farooq N. Prevalence of vitamin D deficiency in general population of Islamabad, Pakistan. Ann Pak Inst Med Sci. (2013) 9:45–7. Available online at: https://www.researchgate.net/publication/269692575_Prevalence_of_Vitamin_D_Deficiency_in_general_population_of_Islamabad_Pakistan

[123] Chlebowski RT Johnson KC Kooperberg C Pettinger M Wactawski-Wende J Rohan T. Calcium plus vitamin D supplementation and the risk of breast cancer. J Natl Cancer Instit. (2008) 100:1581–91. 10.1093/jnci/djn360 19001601 PMC2673920

[124] Tworoger SS Lee I-M Buring JE Rosner B Hollis BW Hankinson SE. Plasma 25-hydroxyvitamin D and 1, 25-dihydroxyvitamin D and risk of incident ovarian cancer. Cancer Epidemiol Prevent Biomark. (2007) 16:783–8. 10.1158/1055-9965.EPI-06-0981 17416771

[125] Bertone-Johnson ER Chen WY Holick MF Hollis BW Colditz GA Willett WC. Plasma 25-hydroxyvitamin D and 1, 25-dihydroxyvitamin D and risk of breast cancer. Cancer Epidemiol Prevent Biomark. (2005) 14:1991–7. 10.1158/1055-9965.EPI-04-0722 16103450

[126] Gupta PC Murti P Bhonsle R. Epidemiology of cancer by tobacco products and the significance of TSNA. Crit Rev Toxicol. (2017) 26:183–98. 10.3109/10408449609017930 8688160

[127] Whitehead TP Havel C Metayer C Benowitz NL Jacob P III. Tobacco alkaloids and tobacco-specific nitrosamines in dust from homes of smokeless tobacco users, active smokers, and nontobacco users. Chem Res Toxicol. (2015) 28:1007–14. 10.1021/acs.chemrestox.5b00040 25794360 PMC4827423

[128] Banerjee SC Ostroff JS Bari S D'Agostino TA Khera M Acharya S. Gutka and Tambaku paan use among South Asian immigrants: a focus group study. J Immigr Minor Health. (2014) 16:531–9. 10.1007/s10903-013-9826-4 23579964 PMC4097304

[129] Shah G Chaturvedi P Vaishampayan S. Arecanut as an emerging etiology of oral cancers in India. Indian J Med Paediatr Oncol. (2012) 33:71. 10.4103/0971-5851.99726 22988348 PMC3439794

[130] Pillai R Balaram P Reddiar KS. Pathogenesis of oral submucous fibrosis. Relationship to risk factors associated with oral cancer. Cancer. (1992) 69:2011–20. 10.1002/1097-0142(19920415)69:8<2011::AID-CNCR2820690802>3.0.CO;2-B 1544110

[131] Thomas G Hashibe M Jacob BJ Ramadas K Mathew B Sankaranarayanan R. Risk factors for multiple oral premalignant lesions. Int J Cancer. (2003) 107:285–91. 10.1002/ijc.11383 12949809

[132] Zhao L Mbulo L Twentyman E Palipudi K King BA. Disparities in smokeless tobacco use in Bangladesh, India, and Pakistan: findings from the global adult tobacco survey, 2014-2017. PLoS ONE. (2021) 16:e0250144. 10.1371/journal.pone.0250144 33886617 PMC8062090

[133] Petti S. Lifestyle risk factors for oral cancer. Oral Oncol. (2009) 45:340–50. 10.1016/j.oraloncology.2008.05.018 18674956

[134] Rao SVK Mejia G Roberts-Thomson K Logan R. Epidemiology of oral cancer in Asia in the past decade-an update (2000-2012). Asian Pac J Cancer Prevent. (2013) 14:5567–77. 10.7314/APJCP.2013.14.10.5567 24289546

[135] García-Martín JM Varela-Centelles P González M Seoane-Romero JM Seoane J García-Pola MJ. Epidemiology of oral cancer. Oral Cancer Detect. (2019) 81–93. 10.1007/978-3-319-61255-3_3

[136] Ferlay J Shin HR Bray F Forman D Mathers C Parkin DM. Estimates of worldwide burden of cancer in 2008: GLOBOCAN 2008. Int J Cancer. (2010) 127:2893–917. 10.1002/ijc.25516 21351269

[137] Qidwai W Saleheen D Saleem S Andrades M Azam I. Are our people health conscious? Results of a patients survey in Karachi, Pakistan. J Ayub Med Coll. (2003) 15:10. 12870308

[138] Mahmood Z. Smoking and chewing habits of people of Karachi−1981. J Pak Med Assoc. (1982) 32:34–7. 6803001

[139] Khawaja M Mazahir S Majeed A Malik F Merchant K Maqsood M. Knowledge, attitude and practices of a Karachi slum population regarding the role of products of betel, areca and smokeless tobacco in the etiology of head & neck cancers. J Pak Med Assoc. (2005) 55:S41.

[140] Shah S Merchant A Luby S Chotani R. Addicted schoolchildren: prevalence and characteristics of areca nut chewers among primary school children in Karachi, Pakistan. J Paediatr Child Health. (2002) 38:507–10. 10.1046/j.1440-1754.2002.00040.x 12354270

[141] Mazahir S Malik R Maqsood M Merchant KA Malik F Majeed A. Socio-demographic correlates of betel, areca and smokeless tobacco use as a high risk behavior for head and neck cancers in a squatter settlement of Karachi, Pakistan. Subst Abuse Treat Prev Policy. (2006) 1:1–6. 10.1186/1747-597X-1-10 16722535 PMC1475829

[142] Javed F Altamash M Klinge B Engström P-E. Periodontal conditions and oral symptoms in gutka-chewers with and without type 2 diabetes. Acta Odontol Scand. (2008) 66:268–73. 10.1080/00016350802286725 18645686

[143] Javed F Chotai M Mehmood A Almas K. Oral mucosal disorders associated with habitual gutka usage: a review. Oral Surg Oral Med Oral Pathol Oral Radiol Endodontol. (2010) 109:857–64. 10.1016/j.tripleo.2009.12.038 20382045

[144] Kaplan S. Novikov l, Modan B. Nutritional factors in the etiology of brain tumors potential role of nitrosamines, fat, and cholesterol. Am J Epidemiol. (1997) 146:832–41. 10.1093/oxfordjournals.aje.a009201 9384204

[145] Lee H-W Park S-H. Weng M-w, Wang H-T, Huang WC, Lepor H, et al. E-cigarette smoke damages DNA and reduces repair activity in mouse lung, heart, and bladder as well as in human lung and bladder cells. Proc Natl Acad Sci USA. (2018) 115:E1560–9. 10.1073/pnas.1718185115 29378943 PMC5816191

[146] Tanuja M. Ghutka and pr-cancerous lesions: An empirical study in ‘twin city' of Odisha. Int J Soc Econom Res. (2018) 8:60–80. 10.5958/2249-6270.2018.00011.9

[147] Ramchandani AG D'Souza AV Borges AM Bhisey RA. Evaluation of carcinogenic/co-carcinogenic activity of a common chewing product, pan masala, in mouse skin, stomach and esophagus. Int J Cancer. (1998) 75:225–32. 10.1002/(SICI)1097-0215(19980119)75:2<225::AID-IJC10>3.0.CO;2-C 9462712

[148] Nigam S Kumar A Sheikh S Saiyed H. Toxicological evaluation of pan masala in pure inbred Swiss mice: a preliminary report on long-term exposure study. Curr Sci. (2001) 80:1306–9. Available online at: http://www.jstor.org/stable/24105045

[149] Duncan RB Briggs M. Treatment of uncomplicated anosmia by vitamin A. Arch Otolaryngol. (1962) 75:116–24. 10.1001/archotol.1962.00740040122008 13888447

[150] Christakos S Li S De La Cruz J Bikle DD. New developments in our understanding of vitamin D metabolism, action and treatment. Metabolism. (2019) 98:112–20. 10.1016/j.metabol.2019.06.010 31226354 PMC6814307

[151] Aguirre M Manzano N Salas Y Angel M Díaz-Couselo FA Zylberman M. Vitamin D deficiency in patients admitted to the general ward with breast, lung, and colorectal cancer in Buenos Aires, Argentina. Arch Osteoporos. (2016) 11:4. 10.1007/s11657-015-0256-x 26732091

[152] Schloss J. Cancer treatment and nutritional deficiencies. In: Erkekoglu P Kocer-Gumusel B editors. Nutritional Deficiency. London: InTech (2016). p. 173–96. 10.5772/63395

[153] Lee E Levine EA Franco VI Allen GO Gong F Zhang Y. Combined genetic and nutritional risk models of triple negative breast cancer. Nutr Cancer. (2014) 66:955–63. 10.1080/01635581.2014.932397 25023197

[154] Xiao X Wu Z-C Chou K-C A. multi-label classifier for predicting the subcellular localization of gram-negative bacterial proteins with both single and multiple sites. PLoS ONE. (2011) 6:e20592. 10.1371/journal.pone.0020592 21698097 PMC3117797

[155] Adaramoye O Akinloye O Olatunji I. Trace elements and vitamin E status in Nigerian patients with prostate cancer. Afr Health Sci. (2010) 10:2. 20811517 PMC2895796

[156] Hurst R Hooper L Norat T Lau R Aune D Greenwood DC. Selenium and prostate cancer: systematic review and meta-analysis. Am J Clin Nutr. (2012) 96:111–22. 10.3945/ajcn.111.033373 22648711

[157] Deschasaux M Souberbielle J-C Latino-Martel P Sutton A Charnaux N Druesne-Pecollo N. Weight status and alcohol intake modify the association between vitamin D and breast cancer risk. J Nutr. (2016) 146:576–85. 10.3945/jn.115.221481 26817718

[158] Cho E Zhang X Townsend MK Selhub J Paul L Rosner B. Unmetabolized folic acid in prediagnostic plasma and the risk for colorectal cancer. J Natl Cancer Inst. (2015) 107:djv260. 10.1093/jnci/djv260 26376686 PMC4715248

[159] Barrett CW Singh K Motley AK Lintel MK Matafonova E Bradley AM. Dietary selenium deficiency exacerbates DSS-induced epithelial injury and AOM/DSS-induced tumorigenesis. PLoS ONE. (2013) 8:e67845. 10.1371/journal.pone.0067845 23861820 PMC3701622

[160] Steinfeld B Scott J Vilander G Marx L Quirk M Lindberg J. The role of lean process improvement in implementation of evidence-based practices in behavioral health care. J Behav Health Serv Res. (2015) 42:504–18. 10.1007/s11414-013-9386-3 24464179

[161] Lippi G Mattiuzzi C Cervellin G. Meat consumption and cancer risk: a critical review of published meta-analyses. Crit Rev Oncol Hematol. (2016) 97:1–14. 10.1016/j.critrevonc.2015.11.008 26633248

[162] Epplein M Burk RF Cai Q Hargreaves MK Blot WJ A. prospective study of plasma Selenoprotein P and lung cancer risk among low-income adults. Cancer Epidemiol Prevent Biomark. (2014) 23:1238–44. 10.1158/1055-9965.EPI-13-1308 24762559 PMC4082447

[163] Xue Y Harris E Wang W Baybutt RC. Vitamin A depletion induced by cigarette smoke is associated with an increase in lung cancer-related markers in rats. J Biomed Sci. (2015) 22:1–9. 10.1186/s12929-015-0189-0 26462767 PMC4605095

[164] Ho E Courtemanche C Ames BN. Zinc deficiency induces oxidative DNA damage and increases p53 expression in human lung fibroblasts. J Nutr. (2003) 133:2543–8. 10.1093/jn/133.8.2543 12888634

[165] Khan RM Iqbal MP. Deficiency of vitamin C in South Asia. Pakistan J Med Sci. (2006) 22:347. Available online at: https://www.pjms.com.pk/issues/julsep06/article/review2.html

[166] Carr AC Rowe S. Factors affecting vitamin C status and prevalence of deficiency: a global health perspective. Nutrients. (2020) 12:1963. 10.3390/nu12071963 32630245 PMC7400679

[167] White R Nonis M Pearson JF Burgess E Morrin HR Pullar JM. Low vitamin C status in patients with Cancer is associated with patient and tumor characteristics. Nutrients. (2020) 12:2338. 10.3390/nu12082338 32764253 PMC7468872

[168] Jacob RA Sotoudeh G. Vitamin C function and status in chronic disease. Nutr Clin Care. (2002) 5:66–74. 10.1046/j.1523-5408.2002.00005.x 12134712

[169] Lechner K Obermeier HL. Cancer-related microangiopathic hemolytic anemia: clinical and laboratory features in 168 reported cases. Medicine. (2012) 91:195–205. 10.1097/MD.0b013e3182603598 22732949

[170] Clark SF. Iron deficiency anemia. Nutr Clin Pract. (2008) 23:128–41. 10.1177/0884533608314536 18390780

[171] Akhtar S Ahmed A Ahmad A Ali Z Riaz M Ismail T. Iron status of the Pakistani population-current issues and strategies. Asia Pac J Clin Nutr. (2013) 22:340–7. Available online at: https://search.informit.org/doi/10.3316/ielapa.507285198519371 23945403 10.6133/apjcn.2013.22.3.17

[172] Nausheen S Habib A Bhura M Rizvi A Shaheen F Begum K. Impact evaluation of the efficacy of different doses of vitamin D supplementation during pregnancy on pregnancy and birth outcomes: a randomised, controlled, dose comparison trial in Pakistan. BMJ Nutr Prevent Health. (2021) 4:425. 10.1136/bmjnph-2021-000304 35028513 PMC8718848

[173] Mustafa G Asadi MA Iqbal I Bashir N. Low vitamin D status in nursing Pakistani mothers in an environment of ample sunshine: a cross-sectional study. BMC Preg Childb. (2018) 18:1–7. 10.1186/s12884-018-2062-0 30373543 PMC6206706

[174] Moorani KN Mustufa MA Hasan SF Kubar N. Vitamin D status in under five children in diverse communities of Karachi. Pakistan J Med Sci. (2019) 35:414. 10.12669/pjms.35.2.680 31086525 PMC6500833

[175] Fund TUNICsE,. National Nutrition Survey 2018: Key Finding Report. (2018). Available online at: https://www.unicef.org/pakistan/media/1871/file/KeyFindings—NationalNutritionSurvey2018.pdf

[176] Griffin TP Wall D Blake L Griffin DG Robinson S Bell M. Higher risk of vitamin D insufficiency/deficiency for rural than urban dwellers. J Steroid Biochem Mol Biol. (2020) 197:105547. 10.1016/j.jsbmb.2019.105547 31756419

[177] Ali A. Current status of malnutrition and stunting in Pakistani children: what needs to be done? J Am Coll Nutr. (2021) 40:180–92. 10.1080/07315724.2020.1750504 32275484

[178] Siegel RL Jakubowski CD Fedewa SA Davis A Azad NS. Colorectal cancer in the young: epidemiology, prevention, management. Am Soc Clin Oncol Educ Book. (2020) 40:e75–88. 10.1200/EDBK_279901 32315236

[179] Reinke S Taylor WR Duda GN Von Haehling S Reinke P Volk H-D. Absolute and functional iron deficiency in professional athletes during training and recovery. Int J Cardiol. (2012) 156:186–91. 10.1016/j.ijcard.2010.10.139 21145121

[180] Saqib M Manzoor F Shahid R Naz S Sharif S. Incidence of anemia among pregnant females of rural areas of punjab. S Asian J Life Sci. (2019) 7:25–8. 10.17582/journal.sajls/2019/7.2.25.282019

[181] Lowe NM Zaman M Moran VH Ohly H Sinclair J Fatima S. Biofortification of wheat with zinc for eliminating deficiency in Pakistan: study protocol for a cluster-randomised, double-blind, controlled effectiveness study (BIZIFED2). BMJ Open. (2020) 10:e039231. 10.1136/bmjopen-2020-039231 33208325 PMC7677336

[182] Bhurgri Y. Karachi cancer registry data–implications for the national cancer control program of pakistan. Asian Pac J Cancer Prev. (2004) 5:77–82. Available online at: http://journal.waocp.org/article_24233.html 15075010

[183] Ashraf MS Jamil A. Cancer care in Pakistan. In: Silbermann M editor. Cancer Care in Countries and Societies in Transition. Cham: Springer (2016). p. 231–45. 10.1007/978-3-319-22912-6_15

[184] Shamsi U. Cancer prevention and control in Pakistan: review of cancer epidemiology and challenges. Liaquat Natl J Prim Care. (2020) 2:34–8. Available online at: https://journals.lnh.edu.pk/lnjpc/pdf/7795eb42-d838-491d-99bb-aade6db21227.pdf

[185] Wazir MS Shaikh BT Ahmed A. National program for family planning and primary health care Pakistan: a SWOT analysis. Reprod Health. (2013) 10:1–7. 10.1186/1742-4755-10-60 24268037 PMC3842797

[186] World Health Organization. Cancer Control: Knowledge Into Action: WHO Guide for Effective Programmes. Policy and Advocacy Module 6. World Health Organization (2008). 24716264

[187] Azhar S Hassali MA Ibrahim MIM Ahmad M Masood I Shafie AA. The role of pharmacists in developing countries: the current scenario in Pakistan. Hum Resour Health. (2009) 7:1–6. 10.1186/1478-4491-7-54 19594916 PMC2714831

[188] Ali A Audi M. The impact of income inequality, environmental degradation and globalization on life expectancy in Pakistan: an empirical analysis (2016). 10.14738/abr.511.3696. Available online at: https://mpra.ub.uni-muenchen.de/71112/

[189] Nishtar S Boerma T Amjad S Alam AY Khalid F. ul Haq I, et al. Pakistan's health system: performance and prospects after the 18th Constitutional Amendment. Lancet. (2013) 381:2193–206. 10.1016/S0140-6736(13)60019-7 23684254

[190] Lee KS Shahidullah A Zaidi ST Patel RP Ming LC Tariq MH. The crux of the medicine prices' controversy in Pakistan. Front Pharmacol. (2017) 8:504. 10.3389/fphar.2017.00504 28824429 PMC5539127

[191] Ahmad N. Status of higher education in nuclear technology in Pakistan (2007). Available online at: https://inis.iaea.org/search/search.aspx?orig_q=RN:40102297

[192] Masood K Ahmad M Zafar J ul Haq M Ashfaq A Zafar H. Assessment of occupational exposure among Pakistani medical staff during 2007-2011. Austral Phys Eng Sci Med. (2012) 35:297-300. 10.1007/s13246-012-0156-y 22847228

[193] Khurshid S. Nuclear medical centres of PAEC. Nucleus. (2020) 42:93–6. Available online at: http://thenucleuspak.org.pk/index.php/Nucleus/article/view/1051

[194] Jamshed A Syed AA Shah MA Jamshed S. Improving cancer care in Pakistan. South Asian J Cancer. (2013) 2:036–7. 10.4103/2278-330X.105892 24455543 PMC3876622

[195] Pakistan CcharC,. Existing Cancer Burden & Treatment Facilities: Cancer Care Hospital Research Centre Pakistan. (2022). Available online at: https://cch-rc.com/why-cancer-hospital/ (accessed April 24, 2022).

[196] Ashraf MS. Pediatric oncology in Pakistan. J Pediatr Hematol Oncol. (2012) 34:S23–5. 10.1097/MPH.0b013e318249abf9 22357147

[197] Iftikhar R Mir MA Moosajee M Rashid K Bokhari SW Abbasi AN. Diagnosis and management of diffuse large B-cell lymphoma: society of medical oncology, Pakistan society of hematology, and Pakistan society of clinical oncology Joint clinical practice guideline. JCO Glob Oncol. (2021) 7:1647–58. 10.1200/GO.21.00320 34898246 PMC9812455

[198] Majeed FA Azeem AR Farhan N. Lung cancer in Pakistan, where do we stand. JPMA. (2019) 69:405–8. Available online at: https://www.jpma.org.pk/article-details/9083 30890835

[199] HealthNet. Cancer Care. Available online at: http://healthnet.com.pk/cc.html (accessed March 23, 2022).

[200] Sciences, PIoM,. About PIMS. Available online at: https://pims.gov.pk/aboutPIMS.htm (accessed March 28, 2022).

[201] Tribune TE,. Free Food Facility Opened at PIMS. (2022). Available online at: https://tribune.com.pk/story/2139614/free-food-facility-opened-pims (accessed April 01, 2022).

[202] Oncology, PSoC,. Mission and Objectives. Available online at: https://psco.com.pk/about/#vision (accessed April 12, 2022).

[203] Centre, SKMCHaR,. About Us. Available online at: https://shaukatkhanum.org.pk/about-us/

[204] Pakistan, SOS,. Surgical Oncology Society Pakistan. Available online at: http://sospk.org/ (accessed April 26, 2022).

[205] Biemar F Foti M. Global progress against cancer-challenges and opportunities. Cancer Biol Med. (2013) 10:183–6. 10.7497/j.issn.2095-3941.2013.04.001 24349827 PMC3860343

[206] Pierre-Louis AM Akala FA Karam HS. Public Health in the Middle East and North Africa: Meeting the Challenges of the Twenty-First Century. World Bank Publications (2004). 10.1596/0-8213-5790-5

[207] Naoum FA. Iron deficiency in cancer patients. Rev Bras Hematol Hemoter. (2016) 38:325–30. 10.1016/j.bjhh.2016.05.009 27863761 PMC5119669

[208] Iniesta RR Rush R Paciarotti I Rhatigan E Brougham F McKenzie J. Systematic review and meta-analysis: prevalence and possible causes of vitamin D deficiency and insufficiency in pediatric cancer patients. Clin Nutr. (2016) 35:95–108. 10.1016/j.clnu.2014.12.023 25638403

[209] Gillberg L Ørskov AD Liu M Harsløf LB Jones PA Grønbæk K.(editors). Vitamin C-A new player in regulation of the cancer epigenome. Seminars Cancer Biol. (2018) 51:59–67. 10.1016/j.semcancer.2017.11.001 29102482

[210] Garland CF Garland FC Gorham ED Lipkin M Newmark H Mohr SB. The role of vitamin D in cancer prevention. Am J Publ Health. (2006) 96:252–61. 10.2105/AJPH.2004.045260 16380576 PMC1470481

[211] Marangoni F Cetin I Verduci E Canzone G Giovannini M Scollo P. Maternal diet and nutrient requirements in pregnancy and breastfeeding. An Italian consensus document. Nutrients. (2016) 8:629. 10.3390/nu8100629 27754423 PMC5084016

[212] Stein AJ. Global impacts of human mineral malnutrition. Plant Soil. (2010) 335:133–54. 10.1007/s11104-009-0228-2

[213] Tippens G. Smokers: Nuisances in Belmont City, California-in their homes, but not on public sidewalks. Minn JL Sci & Tech. (2008) 10:413. Available online at: https://heinonline.org/HOL/LandingPage?handle=hein.journals/mipr10&div=15&id=&page=

[214] Abbasi AS Akhter W Umar S. Ethical issues in advertising in Pakistan: an Islamic perspective. World Appl Sci J. (2011) 13:444–52. Available online at: https://papers.ssrn.com/sol3/papers.cfm?abstract_id=1866800

[215] Wakefield M Morley C Horan JK Cummings KM. The cigarette pack as image: new evidence from tobacco industry documents. Tob Control. (2002) 11:i73–80. 10.1136/tc.11.suppl_1.i73 11893817 PMC1766062

[216] Gilani SI Leon DA. Prevalence and sociodemographic determinants of tobacco use among adults in Pakistan: findings of a nationwide survey conducted in 2012. Popul Health Metr. (2013) 11:1–11. 10.1186/1478-7954-11-16 24004968 PMC3850735

[217] Organization TCL. The Cigarettes (Printing of Warning) Ordinance. Available online at: https://www.tobaccocontrollaws.org/files/live/Pakistan/Pakistan%20%20-%201979%20Ordinance%20.pdf

[218] Organization TCL. Statutory Notifications Containing Rules and Orders issued by all Ministries and Divisions of the Government of Pakistan and their Attached and Subordinate Offices and the Supreme Court of Pakistan. Available online at: https://www.tobaccocontrollaws.org/files/live/Pakistan/Pakistan%20-%20SRO%2086%28KE%29_2009%2C%2087%28KE%29_2009%20-%20national.pdf

[219] Government Government of Pakistan Ministry of Finance EA Statistics Revenue. Notifications (CUSTOMS). Available online at: https://download1.fbr.gov.pk/SROs/201310291510470377SRO693(I)200629oct2013.pdf

[220] Ministry of Health GoP. Prohibition of Smoking and Protection of Non-Smokers Health Ordinance. (2002). Available online at: http://www.tcc.gov.pk/Downloads/Prohibition%20of%20Smoking%20and%20Protection%20of%20Non-Smokers%20Ordinance%20%202002.pdf (accessed April 22, 2022).

[221] Health GoPMo. Notification. Available online at: https://www.tobaccocontrollaws.org/files/live/Pakistan/Pakistan%20-%20SRO%20655%28I%29_2003%20-%20national.pdf (accessed May 01, 2022).

